# Adverse neonatal outcomes of adolescent pregnancy in Northwest Ethiopia

**DOI:** 10.1371/journal.pone.0218259

**Published:** 2019-06-13

**Authors:** Getachew Mullu Kassa, A. O. Arowojolu, A. A. Odukogbe, Alemayehu Worku Yalew

**Affiliations:** 1 Pan African University Life and Earth Sciences Institutes, Department of Obstetrics and Gynaecology, College of Medicine, University of Ibadan, Ibadan, Nigeria; 2 College of Health Sciences, Debre Markos University, Debre Markos, Ethiopia; 3 Department of Obstetrics and Gynaecology, College of Medicine, University College Hospital, University of Ibadan, Ibadan, Nigeria; 4 School of Public Health, College of Health Sciences, Addis Ababa University, Addis Ababa, Ethiopia; University for Development Studies, GHANA

## Abstract

**Background:**

Adolescents have physical, social and psychological characteristics that are different from adults. Adolescent pregnancy results in pregnancy and childbirth complications- an area neglected in developing countries like Ethiopia. This study, therefore, was conducted to assess the adverse neonatal outcomes of adolescent pregnancy in Northwest Ethiopia.

**Methods:**

Institutional-based study was conducted in East Gojjam zone, Northwest Ethiopia. A total of 374 adolescent (15–19 years) and 760 adult (20–34 years) women were included in this study. Data were collected among women who came to randomly selected health facilities in East Gojjam zone. Data were collected by trained research assistants using a structured data collection questionnaire. Descriptive statistics, chi-square test, and Student's t-tests were utilized. Bivariate and multivariable logistic regression analysis were employed to adjust for confounding factors of adverse neonatal outcomes. Statistical significance was declared when the *p-value* was less than 0.05.

**Results:**

Higher proportion of adolescent than adult women were from rural area (57.2% vs 44.7%), were not married (5.1% vs 1.7%), were pregnant for the first time (91.7% vs 34.1%), didn’t attend antenatal care (ANC) follow-up (12% vs 4.5%), and had late initiation of ANC follow-up. After adjusting for known confounding factors, the odds of low birth weight (LBW) was higher among adolescents than adult women (AOR 2.14; 95% CI, 1.36, 3.36, *p-value = 0*.*001*). Similarly, the odds of preterm birth was higher among adolescents than adult women (AOR 1.65; 95% CI, 1.09, 2.49, *p-value* = *0*.*017*). There was no statistically significant difference in the rate of low Apgar score at first and five minutes after birth and neonatal Intensive Care Unit (ICU) admission between babies born from adolescent and adult women.

**Conclusions:**

Adolescent women were less likely to receive ANC service. Babies born from adolescent women are at higher odds of adverse neonatal outcomes like LBW and preterm birth than babies born from adult women. Use of community- and health facility-based intervention programs that can prevent adolescent pregnancy and reduce adverse neonatal outcomes among adolescent girls is recommended.

## Introduction

Adolescent pregnancy is defined as pregnancy that occurs among adolescents aged 10–19 [1, 2]. The rate of pregnancy among adolescents is increasing, especially in developing countries, with higher adverse health outcomes [3]. More than 11 percent of births globally were because of adolescent mothers [4, 5]. Previous studies have shown that adolescent pregnancy is associated with physical, social problems [2], and affects the economic status of girls, their families, and countries [2, 6].

More than one fourth (27%) of women aged 20–24 in developing countries (approximately 12 million) start childbearing at an early age (less than 18 years old) [7]. The recent World Health Organization (WHO) estimate showed that the rate of adolescent pregnancy will grow by the end of 2030, and a major increase in adolescent pregnancy is projected to be in Africa [8].

Adolescent pregnancy is a major public health problem, particularly in Sub Saharan African countries [9]. Problems associated with adolescent pregnancy were considered as the leading causes of death among adolescents aged 16–19 [6]. It is one of the leading causes of maternal and child morbidity and mortality [10, 11], and the main reason for poor health and poverty in low and middle-income countries [8]. Moreover, it is associated with increased risks of adverse pregnancy and childbirth outcomes compared to non-adolescent women [9]. For example, mothers aged 10 to 14 years were five times at higher risk of death than mothers aged 20 to 24 due to pregnancy and childbirth complications [12], attributing to more than 70,000 adolescent girls death every year [12].

Ethiopia is the second most populous country in Africa characterized by high population growth, 2.5% annually [13]. The fertility rate was 5 children per a woman, and most of the population are young people. For example, in 2016, more than one-third (41.6%) of the total population in Ethiopia was under age 15 [14], and 13% of women aged 15–19 have already begun childbearing [15]. Despite the efforts made by the Ethiopian Ministry of Health (MoH) and other stakeholders, the adolescent pregnancy rate is still one of the highest in Africa (72 births per 1000 adolescent girls aged 15–19 years old) [16]. The Ethiopian Demographic and Health Survey (EDHS) report also showed that the percentage of adolescents who started childbearing was high, 12.4% in 2011 and 12.5% in 2016 [15, 17]. A recent study conducted among high school students in Arba Minch town, Southern Ethiopia also showed adolescent pregnancy prevalence of 7.7% [18].

Previous reports have shown a conflicting idea in terms of the adverse neonatal outcomes of adolescent pregnancy. For example, a study conducted in Russia showed that there is a lower risk of adverse pregnancy outcomes like low birth weight (LBW), 5-minute Apgar score, and neonatal admission compared to adult women [5]. A study conducted in Boston city hospital showed that younger age at pregnancy (less than 16 years old) doesn’t predict the likelihood of poor neonatal outcomes (LBW and preterm birth) [19]. In addition, other studies showed a non-significant association between younger maternal age with risk of adverse perinatal outcomes like stillbirth [20], LBW, and preterm birth [21]. On the other hand, studies conducted in Cameroon [22], and low-middle income countries [23] showed a higher risk of LBW and preterm birth [22] among babies born from adolescent compared to adult women. Some of the reasons for such difference, among others, can be the difference in the age group (among the study and control group), the difference in the definition of the outcome variables, and the use or non-use of variables to adjust the confounders for the adverse neonatal outcomes [5]. Moreover, maternal and neonatal mortality is an important reproductive health indicator that can show the health care delivery system of a country. Due to the relationship between such indicators and adolescent pregnancy, the rate of pregnancy among young women (15–19 years old) is considered an important health care indicator of a country [4].

Despite the high number of young people in Ethiopia, the research focus given to these population is limited. Most of the studies conducted in Ethiopia on adolescent health were aimed to estimate the prevalence and determinants of adolescent pregnancy and other sexual and reproductive health (SRH) problems [16, 18, 24, 25]. However, there are limited studies [26, 27] conducted to assess the adverse neonatal outcomes of adolescent pregnancy in Ethiopia, and these studies used secondary data to measure the outcomes, which resulted in limited number of variables to adjust confounding factors. Therefore, this study was conducted to identify the adverse neonatal outcomes of adolescent pregnancy in northwest Ethiopia, and helps to design methods that can reduce problems associated with adolescent pregnancy.

## Materials and methods

### Study area and period

This study was conducted in East Gojjam zone, Amhara region, Ethiopia. According to the 2017 population projection estimate, almost one-fourth of the population in Amhara region were adolescents aged 10–19 years old, of which 49.5% were females [28]. The 2007 Central Statistical Agency (CSA) of Ethiopia report showed that 562,389 of inhabitants in East Gojjam zone were adolescents aged 10 to 19 years old, among which 277,969 (49.4%) were females [29]. According to the 2016/17 annual report of East Gojjam zone health office, there were 19 districts, 102 health centers, eight primary hospitals and one referral hospital in the study area [30]. This study was conducted from January 3/2018 to October 26/2018.

### Study population and eligibility criteria

All pregnant women within the age range of 10 to 34 years old who visited the randomly selected public health institutions for delivery service were included in this study. The study population group were categorized into the adolescent (10–19 years old) and adult women (20–34 years old). Pregnant women with multiple pregnancies (more than one) were excluded from the study since it can influence the neonatal outcomes [31, 32].

### Sample size and sampling procedure

Double population proportion formula was used to calculate the sample size of this study [33]. The following assumptions were considered: two-sided confidence level of 95%, 80% power, the ratio of adult to adolescent women of 2 to 1, and a non-response rate of 10%. This study also considered a design effect of 2, since the multistage sampling technique was used. The proportion of maternal and perinatal outcomes among adolescents and adult women was taken from previously conducted studies [27, 34–36], and the outcome variable which resulted in a maximum sample size was selected. Accordingly, low Apgar score at first and fifth minute from a study conducted in Addis Ababa, Ethiopia [27] resulted in a final sample size of 1254 mothers (418 adolescents and 836 adult women).

This study used a multistage sampling technique to select a representative sample of health facilities in East Gojjam zone. First, random selection of the districts was made from all districts in the zone. From the total of 19 districts in the zone, 7 (37%) of the districts and 12 health facilities were randomly selected. The proportional sample was allocated to the selected health institutions in the study area based on their previous annual client flow.

### Study instrument, quality assurance, and pretest

A structured interview questionnaire was used for data collection. The data collection tool was prepared after reviewing several research articles, Demographic and Health Survey (DHS) documents, recommendations and published works on adolescent pregnancy and related topics [17, 37–49]. Qualified research assistants were involved in the data collection and supervision. Training was provided for data collectors on the objectives of the study, data collection methods, ethical issues, and contents of the questionnaire. Before the actual data collection period, a pretest of the data collection instrument was conducted. Correction to the data collection instrument was made after the pretest.

### Data collection procedures

Study participants were enrolled in the study during the labor and delivery. All laboring women who came to the randomly selected health facilities for delivery service were assessed for eligibility and interviewed until proportionally distributed sample size for both groups of the population was met. Data on sociodemographic, obstetric, and newborn outcomes were collected from the study participants after admission into the maternity ward for labor and childbirth.

### Definition of outcomes

Adverse neonatal outcome was defined as the occurrence of LBW, preterm delivery, low Apgar score at first and fifth minutes after birth, or severe neonatal conditions. LBW is defined as the delivery of a live infant whose birth weight is less than 2500 grams [50, 51]. Preterm delivery is defined as the delivery of the baby less than 37 weeks of gestation [51]. The severe neonatal condition is defined as neonates presenting with any of the following conditions: birth weight less than 1500 grams, gestational age less than 32 weeks or Apgar score at 5 minutes less than 7) [52, 53].

### Data management and analysis methods

After data collection, data were entered using EpiData (Denmark) version 3.1 software. Data analysis was conducted using Statistical Package for the Social Sciences (IBM SPSS) version 25, and R-(version 3.5.1) software. Descriptive statistics like frequencies and summary statistics (mean, standard deviation (SD), and percentage) were used to describe the study population in relation to socio-demographic and other relevant variables. Categorical data between adolescent and adult women were compared using the chi-square test, and independent t-test was used for comparison of the mean difference of continuous variables between the two population groups.

Bivariate and multivariate logistic regression analysis was conducted to identify factors associated with adverse neonatal outcomes. The bivariable logistic regression was conducted to assess to association of each independent variables with the adverse neonatal outcomes. Then, variables with p-value less than 0.2 in the bivariate logistic regression analysis were entered into the multivariable logistic regression analysis to control for confounding factors and identify the factors associated with adverse neonatal outcomes. The variables included in the multivariable logistic regression model include: sociodemographic, economic and obstetric factors like: maternal age (adolescent vs adult), residence, school attendance, marital status, wealth status, educational status of the father and the mother, anemia, iron-folic acid supplementation during current pregnancy, ANC attendance, partner involvement in ANC, experience of at least one form of gender based violence (physical, sexual or psychological violence) during the current pregnancy, and preeclampsia variables. Separate logistic regression analysis models covering the different adverse neonatal outcomes (low birth weight, preterm birth, low Apgar score at birth and five minutes, and neonatal ICU admission rate) were tested and presented. The Adjusted Odds Ratio (AOR) and 95% Confidence Interval (CI) of the regression model was used to determine the association of adolescence pregnancy and other explanatory variables with different adverse neonatal outcomes. Statistical significance was declared when the *p-value* is less than 0.05. In addition, the percentage of LBW and preterm birth was presented by the residence and ANC use of study participants.

### Ethics considerations

The study was approved by the Institute for Advanced Medical Research and Training (IAMRAT), College of Medicine, University of Ibadan, Ibadan, Nigeria with the I/UCH EC Registration Number of NHREC/05/01/2008a and UI/UCH Ethics Committee assigned number of UI/EC/17/0440. The study was also approved by Amhara Public Health Institute and Debre Markos University. Informed consent was obtained from study participants before data collection. The collected information during the course of the research was treated with the utmost confidentiality.

## Results

### Sociodemographic characteristics

This study was conducted among 1134 study participants, accounting for 90.4% of the total sample size. A total of 374 (response rate = 89.5%) adolescents and 760 (response rate = 90.9%) adult women completed the study. The mean age ± standard deviation (SD) of adolescent women included in the study was 18.4 (± 0.8) years (range from 15 to 19 years old). For adult women, the mean age (± SD) of participants was 27 (± 3.9) years, ranging from 20 to 34 years old. More than half (57.2%) of adolescent and 44.7% of adult women were rural residents. More than two third (72.2%) of adolescent and 56.7% of adult women attended school. From educated women, 70% of adolescents and 40% of adult women attended primary education, while 2% and 17.2% attended higher education. Almost all women were Amhara by ethnicity in both groups of the population. The majority (93.3% and 94.9%) of the adolescent and adult women were Orthodox Christian religion followers. Higher proportion of adolescents than adult women (5.1% vs 1.7%, *p-value < 0*.*001*) were not married **(Table 1)**.

**Table 1:** Sociodemographic characteristics of women who gave birth at public health facilities in East Gojjam zone, Northwest Ethiopia, 2018.

Variables		Adolescents (15–19 years old) n (%)	Adults (20–34 years old) n (%)	*p-value*
Residence	Urban	160 (42.8)	420 (55.3)	*< 0*.*001*
	Rural	214 (57.2)	340 (44.7)	
Ever attended school	Yes	270 (72.2)	431 (56.7)	*< 0*.*001*
	No	104 (27.8)	329 (43.3)	
Educational level if attended	Primary education	189 (70)	171 (39.7)	*< 0*.*001*
	Secondary education	62 (23)	141 (32.7)	
	Technical/vocational	14 (5.2)	45 (10.4)	
	Higher education	5 (1.9)	74 (17.2)	
Ethnicity	Amhara	374 (100)	757 (99.6)	*0*.*687*
	Others*	0	3 (0.4)	
Religion	Orthodox Christian	349 (93.3)	721 (94.9)	*0*.*470*
	Muslim	22 (5.9)	33 (4.3)	
	Others**	3 (0.8)	6 (0.8)	
Educational status of the father	Unable to read and write	259 (69.3)	517 (68)	*0*.*345*
	Can read and write	88 (23.5)	193 (25.4)	
	Grade 1–6	5 (1.3)	8 (1.1)	
	Grade 7–12	5 (1.3)	20 (2.6)	
	College level and above	17 (4.5)	22 (2.9)	
Educational status of the mother	Unable to read and write	297 (79.4)	629 (82.8)	*0*.*731*
	Can read and write	59 (15.8)	103 (13.6)	
	Grade 1–6	6 (1.6)	10 (1.3)	
	Grade 7–12	6 (1.6)	9 (1.2)	
	College level and above	6 (1.6)	9 (1.2)	
Occupational status of the father	Daily laborer	19 (5.1)	14 (1.8)	*0*.*004*
	Farmer	268 (71.7)	572 (75.3)	
	Civil servant	24 (6.4)	48 (6.3)	
	Employed in private business	16 (4.3)	23 (3)	
	Has private business	37 (9.9)	59 (7.8)	
	Others^a^	10 (2.7)	44 (5.8)	
Occupational status of the mother	Daily laborer	11 (2.9)	6 (0.8)	*0*.*054*
	Farmer	271 (72.5)	581 (76.4)	
	Civil servant	6 (1.6)	14 (1.8)	
	Employed in private business	11 (2.9)	17 (2.2)	
	Has private business	31 (8.3)	46 (6.1)	
	Others^b^	44 (11.8)	96 (12.6)	
Marital status	Currently married	355 (94.9)	747 (98.3)	*< 0*.*001*
	Yes, living with a man	10 (2.7)	2 (0.3)	
	No, not in union	9 (2.4)	11 (1.4)	
Wealth quantile	Lowest	149 (19.6)	85 (22.7)	*0*.*534*
	Second	148 (19.5)	69 (18.4)	
	Middle	165 (21.7)	69 (18.4)	
	Fourth	150 (19.7)	71 (19)	
	Highest	148 (19.5)	80 (21.4)	

*Tigray, Oromo, SNNPR

**Catholic, protestant

^*a*^ priest, retired, or dead

^*b*^ house wife or dead

### Obstetric characteristics

The majority (91.7%) of adolescent and 259 (34.1%) of adult women were pregnant for the first time. The mean age ± SD of first pregnancy among adolescents was 16.1 ± 1.5 and adults was 20.2 ± 3.3 years. The mean and SD for a previous number of pregnancies was 2.4 ± 1.6 for overall (1.3 ± 0.5 among adolescent women and 2.5 ± 1.6 for adult women). More adolescents than older women who had previous history of pregnancy didn’t give birth in a health facility (51.6% vs 31.1%, *p-value < 0*.*018*). Similarly, significantly lower proportion of adolescent women had ANC follow-up during the current pregnancy than older women (88% vs 95.5%, *p-value < 0*.*0001*), and received iron and folic acid supplementation during current pregnancy (68.4% vs 77.8%, *p-value = 0*.*001*). The mean ± SD of gestational age during antenatal care booking for adolescent women was significantly later than older women (18 ± 8 vs 15.3 ± 6.6, *p-value < 0*.*0001*) (**Table 2**).

**Table 2:** Obstetric characteristics of women who gave birth at public health facilities in East Gojjam zone, northwest Ethiopia, 2018.

Variables		Adolescents (15–19 years old) n (%)	Adults (20–34 years old) n (%)	*P-value*
Previous pregnancy	No	343 (91.7)	259 (34.1)	*< 0*.*001*
	Yes	31 (8.3)	501 (65.9)	
Age at first pregnancy (in years)	(mean ± SD)	16.1 ± 1.5	20.2 ± 3.3	*< 0*.*001*
Total number of previous pregnancies	(mean ± SD)	1.3 ± 0.5	2.5 ± 1.6	*< 0*.*001*
Previous history of institutional delivery	No	16 (51.6)	156 (31.1)	*0*.*018*
	Yes	15 (48.4)	345 (68.9)	
Attend ANC checkup for the current pregnancy	No	45 (12)	34 (4.5)	*< 0*.*0001*
	Yes	329 (88)	726 (95.5)	
Time of first ANC booking (in weeks)	(mean ± SD)	18 ± 8	15.3 ± 6.6	*< 0*.*0001*
Number of ANC visits	(mean + SD)	3.26 ± 1.24	3.54 ± 1.5	*0*.*003*
Type of health facility for ANC follow-up*	Hospital	56 (17.3)	173 (24.1)	*0*.*014*
	Health center	273 (84.3)	563 (78.3)	*0*.*026*
	Private clinic or hospital	14 (4.3)	41 (5.7)	*0*.*356*
Received iron and folic acid supplementation during current pregnancy	No	118 (31.6)	169 (22.2)	*0*.*001*
	Yes	256 (68.4)	591 (77.8)	
Tetanus toxoid vaccination during current pregnancy	No	75 (20.1)	122 (16.1)	*0*.*095*
	Yes	299 (79.9)	638 (83.9)	
Partner came to health facility for ANC purpose during the current pregnancy	No	178 (52.8)	314 (42.5)	*0*.*002*
	Yes	159 (47.2)	425 (57.5)	

*multiple responses possible

### Newborn characteristics

Almost half (47.6%) of newborn babies among adolescent and 44% among adult women were females, *p-value = 0*.*246*. There were three newborn death cases among adolescents and 15 cases among adult women (*p-value = 0*.*138*). The median time for breastfeeding initiation for both adolescent and adult women was 60 minutes. Almost similar proportion of adolescent and adult women identified the type of breastfeeding (98.7% vs 98.8%, *p-value = 0*.*843*) and started breastfeeding within one hour (84.8% vs 87.4%, *p-value = 0*.*227*), but the difference was not statistically significant. Kangaroo Mother Care (KMC) was practiced by 91.3% of adolescent and 85% adult women who had LBW babies.

Additionally, 21 (5.7%) of newborn babies from adolescent and 41 (5.5%) from adult women were admitted to the Intensive Care Unit (ICU), *p-value = 0*.*914*. The main reason for ICU admission for adolescents and adult women were prematurity (61.9% vs 42.5%) and asphyxia (23.8% vs 32.5%), *p-value = 0*.*463*. The main fetal presentation was cephalic for adolescent (97.9%) and adult women (95.3%), *p-value = 0*.*098*. The occipito-anterior (91.7% vs 87.2%) and occipito-posterior position (3.8% vs 2.4%) were the main positions for babies of adolescents than adult women, respectively (*p-value = 0*.*012)* (**Table 3**).

**Table 3:** Newborn characteristics of women who gave birth at public health facilities in East Gojjam zone, Northwest Ethiopia, 2018.

Variables		Adolescents (15–19 years old) n (%)	Adults (20–34 years old) n (%)	*p-value*
Sex of the newborn baby	Male	196 (52.4)	426 (56.1)	*0*.*246*
	Female	178 (47.6)	334 (43.9)	
Newborn outcome	Alive	371 (99.2)	745 (98)	*0*.*138*
	Dead	3 (0.8)	15 (2)	
Birthweight of the baby (in grams)	< = 2499	46 (12.4)	42 (5.6)	*< 0*.*0001*
	2500–3999	324 (87.3)	699 (93.8)	
	> = 4000	1 (0.3)	4 (0.5)	
APGAR score at 1^st^ minute after birth	<6	22 (5.9)	47 (6.3)	*0*.*805*
	7–10	349 (94.1)	698 (93.7)	
APGAR score at 5 minutes after birth	<6	5 (1.3)	13 (1.7)	*0*.*620*
	7–10	366 (98.7)	732 (98.3)	
Gestational age at birth	Less than 37 weeks	51 (14.1)	61 (8.1)	*0*.*002*
	> = 37 weeks	311 (85.9)	691 (91.9)	
Mother identified type of breast feeding	Yes	366 (98.7)	736 (98.8)	*0*.*843*
	No	5 (1.3)	9 (1.2)	
Breast feeding initiation	Within 1 hour	313 (84.8)	648 (87.4)	*0*.*227*
	After 1 hour	56 (15.2)	93 (12.6)	
Newborn admitted to NICU	No	350 (94.3)	704 (94.5)	*0*.*914*
	Yes	21 (5.7)	41 (5.5)	
Reason for NICU	Prematurity	13 (61.9)	17 (42.5)	*0*.*463*
	Infection	1 (4.8)	5 (12.5)	
	Asphyxia	5 (23.8)	13 (32.5)	
	Other*	2 (9.5)	6 (14.6)	
Treatment given to the newborn baby	Yes	71 (19.1)	88 (11.8)	*0*.*001*
	No	300 (80.9)	657 (88.2)	
Fetal presentation	Cephalic	365 (97.9)	724 (95.3)	*0*.*098*
	Breech	7 (1.9)	29 (3.8)	
	Others**	1 (0.3)	7 (0.9)	
Fetal position	Occipito anterior	341 (91.7)	656 (87.2)	*0*.*012*
	Occipito posterior	14 (3.8)	18 (2.4)	
	Right ocipito lateral	3 (0.8)	24 (3.2)	
	Left ocipito lateral	6 (1.6)	24 (3.2)	
	Others***	8 (2.2)	30 (4)	

*unable to breast feed, cephalohematoma, jaundice, respiratory distress

**shoulder, face, brow

***frank breech, hand prolapse, mento-anterior, unspecified

### Adverse neonatal outcomes

#### Low birth weight

The mean birth weight of babies born among adolescent women was 2911.6 grams and was 3050 grams among adult women *(t-test = -138*.*4*, *p-value < 0*.*001)*. The average number of birth weight was 138.4 grams lower among babies born from adolescent compared to babies from adult women. After category, 12.4% of adolescent women had LBW babies and it was 5.6% among adult women, *p-value < 0*.*0001*.

The bivariate logistic regression analysis for association of LBW and different sociodemographic, economic and obstetric factors showed that variables like residence, school attendance, wealth status, educational status of the father, educational status of the mother, malaria attack during current pregnancy, gender-based violence during current pregnancy, and anemia were not statistically significant with LBW. However, variables like: adolescent pregnancy, ANC attendance during current pregnancy, Iron-folic acid supplementation during current pregnancy, and preeclampsia variables were significantly associated with LBW. After multivariable logistic regression analysis, adolescent pregnancy (AOR 2.14; 95% CI, 1.36, 3.36, *p-value* = 0.001) and preeclampsia (AOR 3.21; 95% CI, 1.45, 7.08, *p-value =* 0.004) were found to be significantly associated with LBW (**Table 4**).

**Table 4:** Determinants of selected poor neonatal outcomes associated with adolescent pregnancy in Northwest Ethiopia, 2018.

Variable		Low birth weight		Preterm birth	
		COR (95% CI)	AOR (95% CI)	COR (95% CI)	AOR (95% CI)
Age of the mother (years)	Adolescents (15–19)	2.36 (1.52, 3.66) ***	2.14 (1.36, 3.36) **	1.86 (1.25, 2.76) **	1.65 (1.09, 2.49) *
	Adults (20–34)	Reference	Reference	Reference	Reference
Residence	Urban	1.49 (0.96, 2.32)	1.23 (0.78, 1.95)	0.87 (0.59, 1.29	0.95 (0.63, 1.43)
	Rural	Reference	Reference	Reference	Reference
Anemia	No	Reference	Reference	Reference	Reference
	Yes	0.66 (0.16, 2.78)	0.69 (0.16, 3.02)	1.05 (0.37, 3.03)	1.13 (0.39, 3.33)
Iron-folic acid supplementation	No	Reference	Reference	Reference	Reference
	Yes	0.56 (0.35, 0.89) *	0.75 (0.43, 1.31)	0.53 (0.35, 0.79) **	0.75 (0.45, 1.25)
GBV during current pregnancy	No	Reference	Reference	Reference	Reference
	Yes	1.13 (0.6, 2.15)	0.85 (0.44, 1.65)	1.09 (0.62, 1.95)	0.85 (0.47, 1.56)
ANC attendance	No	Reference	Reference	Reference	Reference
	Yes	0.38 (0.19, 0.72) **	0.58 (0.27, 1.27)	0.29 (0.17, 0.51) ***	0.41 (0.21, 0.83) *
Preeclampsia	No	Reference	Reference	Reference	Reference
	Yes	3.22 (1.49, 6.93) **	3.21 (1.45, 7.08) **	2.63 (1.27, 5.46) **	2.59 (1.22, 5.48) *

*significant at p < 0.05

**significant at p < 0.01

***significant at p < 0.001

AOR: Adjusted Odds Ratio; COR: Crude Odds Ratio; CI: Confidence Interval

**Fig 1:**
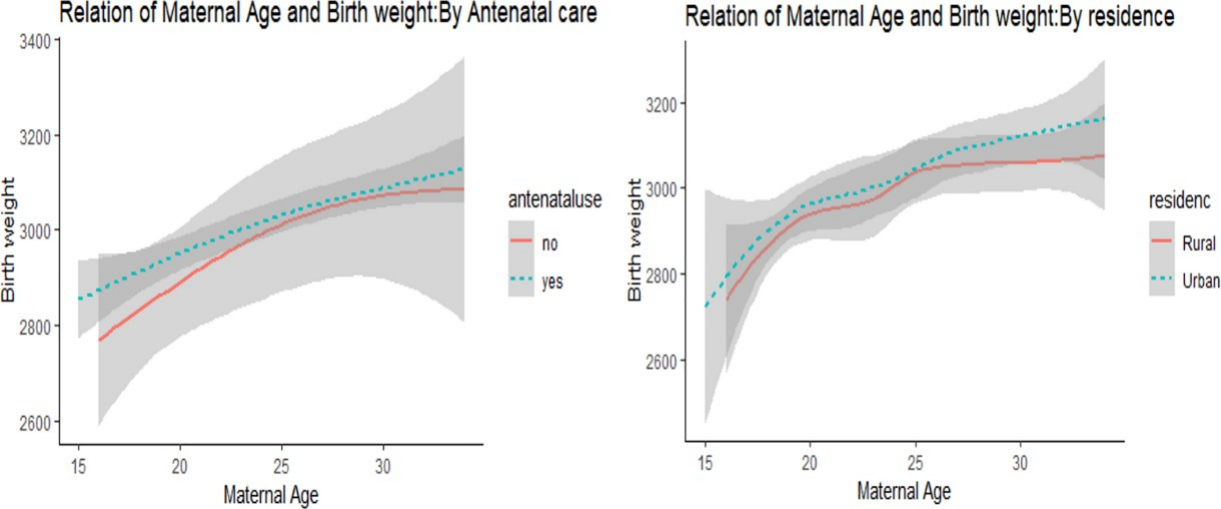
The relationship of women’s age and birth weight by antenatal use and residence among women who gave birth in public health facilities, East Gojjam zone, Northwest Ethiopia, 2018.

Further stratified analysis of birth weight by residence and ANC follow up was also conducted. Accordingly, 29 (13.7%) of babies born from adolescent women who were from the rural area had low birth weight compared to 17 (10.7%) of babies from urban adolescent women. Additionally, 22 (6.6%) of newborn babies born from adult women in the rural area had low birth weight compared to 20 (4.8%) in the urban area. In addition, analysis of birth weight by antenatal follow up showed a higher percentage of LBW among adolescents who had no ANC follow up 11 (26.2%) compared to adults who had no ANC follow up 2 (5.9%). The percentage of LBW was also high for adolescent women who had ANC follow up 35 (10.7%) than babies of adult women who had ANC follow up 40 (5.7%) **(Fig 1)**. Moreover, after adjusting the effect of residence and ANC follow up, the odds of LBW was still significantly higher among adolescent women than among adult women (**Table 4**).

#### Preterm birth

**Fig 2:**
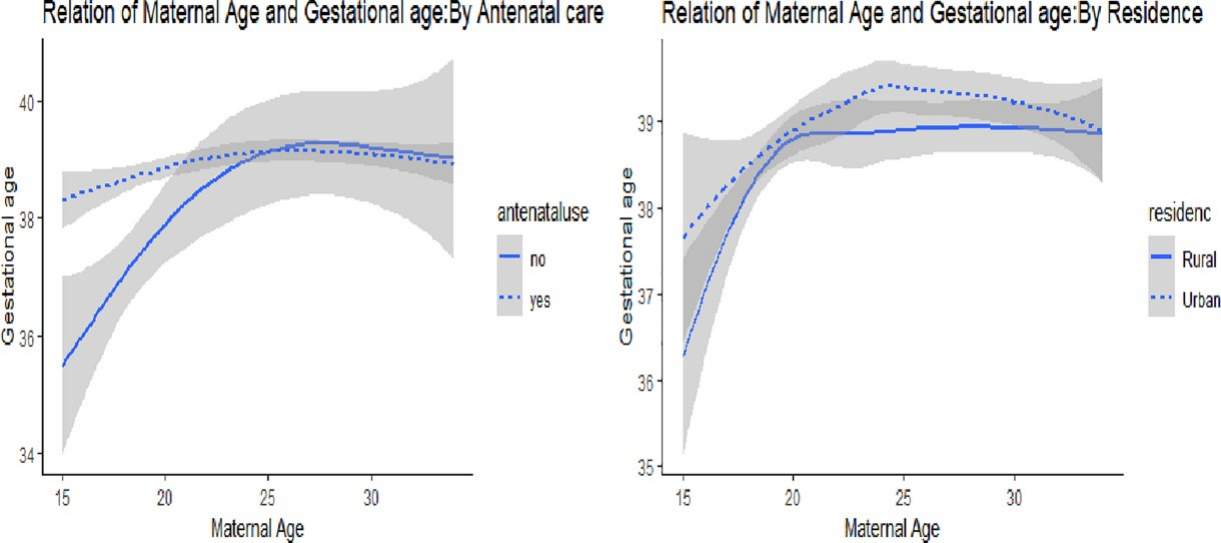
The relationship of women’s age and gestational age at birth by antenatal use and residence among women who gave birth in public health facilities, East Gojjam zone, Northwest Ethiopia, 2018.

The percentage of preterm birth was higher 51 (14.1%) for newborn babies born from adolescent women compared to 61 (8.1%) among babies of adult women. Relative to adolescents in the urban area, a higher percentage of newborn babies born from adolescents in the rural area had preterm birth (13.5% vs 14.6%). In addition, a higher proportion of adolescent than adult women who didn’t attend antenatal follow-up had preterm birth (40.5% vs 6.1%) (**Fig 2**).

The bivariate logistic regression analysis showed that variables like residence, school attendance, previous pregnancy, marital wealth status, educational status of the father, educational status of the mother, GBV during current pregnancy, and anemia were not statistically significant with preterm birth. But, variables like adolescent pregnancy, Iron-folic acid supplementation during current pregnancy, ANC attendance, and preeclampsia were significantly associated with preterm birth. After multivariate logistic regression analysis, variables like: adolescent pregnancy (AOR 1.65; 95% CI, 1.09, 2.49, *p-value* = 0.017), ANC attendance during current pregnancy (AOR 0.41; 95% CI, 0.21, 0.83, *p-value* = 0.013), and preeclampsia (AOR 2.59; 95% CI, 1.22, 5.48, *p-value* = 0.013) were significantly associated with preterm birth (**Table 4**).

#### Apgar score at first and five minutes after birth

Twenty-two (5.9%) of babies born from adolescent women had Apgar score at birth less than 7 compared to 47 (6.3%) among adult women (**Table 3**). The mean Apgar score at first minute after birth for babies born from adolescent and adult women was almost similar (7.7 vs 7.8). The average Apgar score was almost 0.1 lower among babies born from adolescents compared to babies born from adult women, but was not statistically significant. Five (1.3%) and 13 (1.7%) of babies born from adolescent and adult women, respectively, had Apgar score less than 7 at five minutes after birth. The bivariate logistic regression analysis showed non-statistically significant difference in the Apgar score at first minute after birth between babies born from adolescent and adult women (COR 0.94; 95% CI, 0.56, 1.59, *p-value = 0*.*805*). In addition, there was non-statistically significant difference of Apgar score at fifth minute after the birth of babies born from adolescent and adult women, (COR 0.77; 95% CI, 0.27, 2.17, *p-value = 0*.*621*).

#### Neonatal admission to ICU and severe neonatal conditions

**Fig 3:**
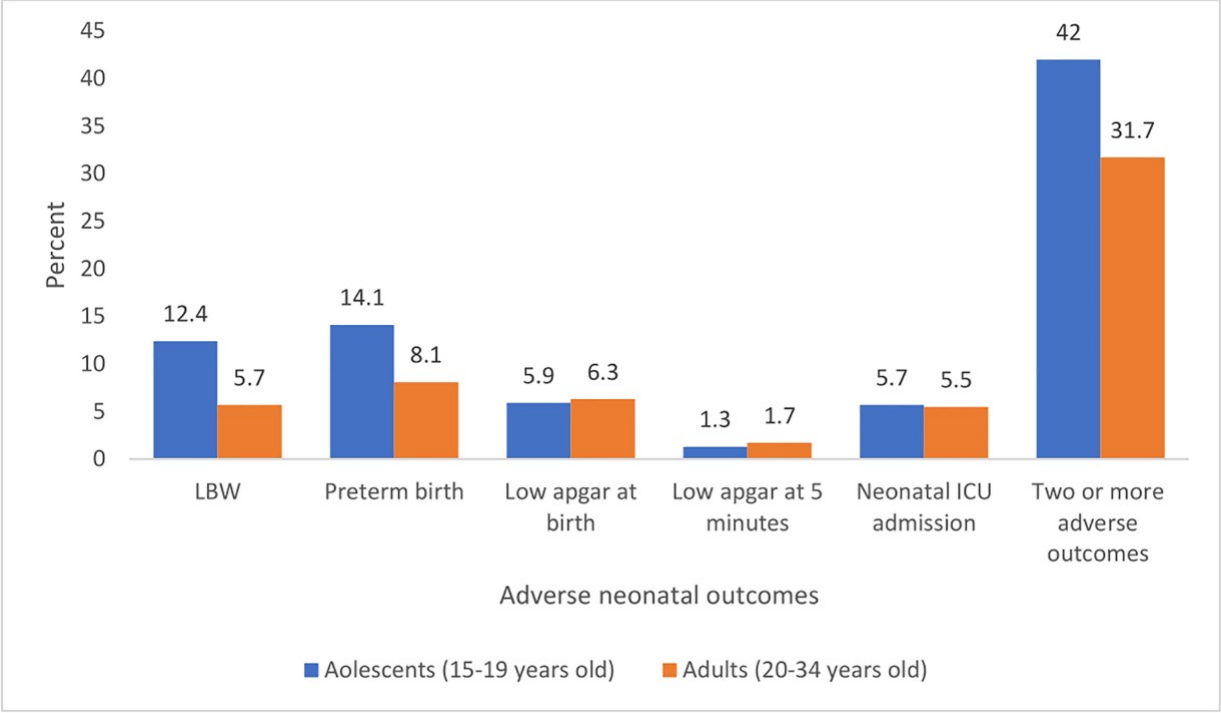
Adverse neonatal outcomes among adolescent and adult women who gave birth in public health facilities, East Gojjam zone, Northwest Ethiopia, 2018.

Six percent of babies born from adolescents were admitted to ICU compared to 5.5% among adult women. The bivariate logistic regression analysis showed that there was non-statistically significant difference in the neonatal admission rate to ICU between babies born from adolescent and adult women (COR 0.94; 95% CI, 0.56, 1.59, *p-value = 0*.*805*). In addition, a summary index was created for the occurrence of at least two or more of adverse neonatal conditions like; LBW, preterm birth, low Apgar score at first or at 5 minutes, or neonatal ICU admission. Accordingly, 34 (42%) of newborn babies of adolescents had at least two or more adverse neonatal conditions compared to 40 (31.7%) in adult women (**Fig 3**).

## Discussion

This study was conducted to assess the adverse neonatal outcomes of adolescent pregnancy in northwest Ethiopia. A significant difference in socio-demographics, obstetric characteristics and newborn outcomes between adolescent and adult women was found. Sociodemographic factors like residence, school attendance, level of education attended, educational status of the father, and marital status were significantly different between adolescent and adult women. A higher proportion of adolescent women had lower mean age at first pregnancy and didn’t attend ANC during pregnancy.

This study adjusted for different known factors to assess the adverse newborn outcomes associated with adolescent pregnancy. After adjustment, relative to babies born from adult women, babies born from adolescent women were at higher odds of LBW (2.14 times) and preterm birth (1.65 times). However, there was no statistically significant difference in the Apgar score value at first and five minutes after birth, ICU admission, severe neonatal conditions, and occurrence of two or more types of adverse neonatal outcomes between newborn babies of adolescent and adult women.

Although the use of ANC is recommended for all pregnant women to reduce pregnancy-related morbidity and mortality [54], this study found significantly higher proportion (12%) of adolescents who didn’t attend ANC follow-up compared to adult women (4.5%). The average gestational age to start antenatal follow-up was almost three weeks late for adolescents than adult women. Previous studies conducted in Slovenia [55], Thailand [56], and Turkey [57] also showed significantly lower antenatal care use among adolescents compared to adult women. This could be because of the difference in educational status between adolescent and adult women [54, 58, 59]. In addition, reproductive health care access for pregnant adolescents is limited in most sub-Saharan African countries [60]. This finding suggests the need to improve access to and quality of ANC and targeted pregnancy-related services for adolescent pregnant women. Strengthening female education and women empowerment is central to improve ANC service use and reduce pregnancy-related complication among adolescents. Moreover, women empowerment is related to family planning use [61] and fertility reduction [62].

Almost one in ten adolescents were pregnant for the second time in this study. Socioeconomic and marital status during first birth affects the occurrence of second-time pregnancy among adolescents in a previous report [63]. Therefore, prevention of early marriage should be one of the main focus of programs which aimed at preventing adolescent pregnancy. Moreover, in most low-income countries, marriage is the main reason for first sexual initiation [64]. Postnatal family planning information and service provision is also central to the prevention of repeated adolescent pregnancy.

The high rate of neonatal complications can be reduced through the use of institutional service delivery [65]. Despite this, home delivery is still a common practice in Ethiopia [65]. This study also found that a significantly higher percentage of adolescents (51.6%) than older women (31.1%) who had previous history of pregnancy didn’t give birth in a health facility. The low institutional delivery service use among adolescent women can be related to the poor knowledge of adolescent women towards skilled delivery service [65]. Therefore, community-based programs that can improve the knowledge and institutional delivery service use of pregnant adolescents is recommended. Strengthening the use of health extension program to improve institutional delivery service use among adolescents is also effective [66].

Although the majority of adolescent (98.7%) and adult women (98.8%) in this study identified the type of breastfeeding before or immediately after childbirth, only 84.8% of adolescent and 87.4% of adult women started breastfeeding within the first one hour after childbirth. This finding is higher compared to previous studies conducted in Dembecha district, Ethiopia (73.1%) [67], and meta-analysis finding in Ethiopia (61.4%) [68]. This may be due to the difference in the study period and the effectiveness of current programs provided by governmental and non-governmental organizations to improve the breastfeeding practice. Moreover, WHO promotes breastfeeding initiation within one hour after childbirth to ensure that neonate receives adequate nutrition and protective antibodies from colostrum [69]. Review of studies showed that early initiation of breastfeeding is associated with reduced neonatal morbidity and mortality [70]. One study conducted in Bahir Dar, Ethiopia also showed a higher risk of neonatal mortality among babies who were breastfed later than one hour after birth [71]. Therefore, programs that can improve the knowledge and practice of adolescent women towards early breastfeeding initiation are recommended.

The mean birth weight of babies born from adolescent women was significantly lower than babies born from adult women. Increased odds (2.14 times) of LBW among adolescent women could be due to the low pregnancy weight gain secondary to the poor nutritional status, and biological immaturity [53, 72]. This is in keeping with other studies conducted in Cameroon [22], Reunion Island (Indian Ocean) [73], Thailand [74], Turkey [57], Washington State, USA [75], and low-middle income countries [23]. One-fifth of all births globally are LBW, with an increased risk of neonatal morbidity and mortality [76]. As a result, the WHO during the sixty-fifth world health assembly (in May 2012) planned to reduce LBW by 30% by the end of 2025 [77]. Therefore, strategies which aim to reduce adolescent pregnancy and immediate management of newborn babies of adolescent women is effective to achieve such target. Preeclampsia was also found to be a factor associated with LBW. This could be because of the effect of high blood pressure on infant in-utero growth, resulting in LBW [78]. This finding is similar with previous study conducted in low- and middle-income countries [79].

Additionally, 91.3% of adolescent women who had LBW practiced Kangaroo Mother Care (KMC) practice. However, this is below to the current guideline on KMC. The WHO recommends a skin-to-skin contact for all infants with LBW, because it was proven to be effective in improving initiation and duration of breastfeeding and for effective thermal control [80]. Therefore, immediate management of LBW through the use of KMC should be strengthened among adolescent women. But, the effect of KMC on infant survival needs further investigation.

Preterm birth was also associated with an increased risk of neonatal mortality [81]. The current study found significantly higher odds (1.65 times) of preterm birth among adolescent compared to adult women. This could be because of the anatomical and physiological immaturity, underweight, poor weight gain during pregnancy, and inadequate ANC among adolescent women [53, 82]. The finding of this study is consistent with previous studies [2, 22, 23, 57, 73–75, 83, 84]. Therefore, intervention programs that focus on prevention of preterm birth and its complications among adolescents through antenatal care follow-up and intrapartum management are recommended. The use of prophylactic antibiotics to prevent neonatal sepsis and medications that can improve fetal lung maturity is also effective in improving outcomes of preterm babies [81, 85]. Such interventions should especially target adolescent women as the risk is more common in younger than adult women.

In addition to younger maternal age, factors like ANC attendance during current pregnancy and preeclampsia were found to be significant factors associated with preterm birth. The higher odds of preterm birth among women who didn’t attend ANC service could be because of the lack of services provided during ANC follow up, like maternal iron-folic acid supplementation, Tetanus Toxoid vaccination, counselling on nutrition and other services. This finding is similar with a study conducted in Belgium, which showed that sufficient and appropriate timing of care during pregnancy is associated with lower risk of preterm birth [86]. It is also similar with a study conducted in Zimbabwe [87]. In addition, babies born from women with preeclampsia were also more likely to be born prematurely. Though the mechanism is not well understood, higher odds of preterm birth among women who have preeclampsia could be because of the effect of high blood pressure resulting in uteroplacental ischemia [88]. The finding is similar with previous studies conducted in New South Wales [78], Kenya [88], and a study conducted in low- and middle-income countries [79].

Neonatal mortality, which is responsible for 38% of under-five mortality globally, is mainly predicted by LBW and prematurity [89, 90]. The perinatal mortality rate in Ethiopia is one of the highest in sub-Saharan Africa countries. For example, one study conducted on the trend of perinatal mortality rate in Ethiopia showed a rate of 90 per 1000 birth in a hospital setting, and 40 per 1000 births in community-based settings [91]. Maternal age less than 18 years old is the main significant factor for the high rate of neonatal mortality in Ethiopia [90]. The rate of perinatal mortality increase by half among adolescent women than adults aged 20 to 29 [92]. Therefore, investment in the design of school and community-based intervention programs aimed at reducing adolescent pregnancy and its adverse neonatal outcomes are needed to reduce the high rate of child morbidity and mortality. It will also help to achieve the Sustainable Development Goal (SDG) target 3.2 which aims to “end preventable deaths of newborn and children under 5 years of age” by 2030 [93].

Several countries used successful programs in reducing adolescent pregnancy, maternal and child morbidity and mortality and improving maternal and child health. The success story of such fast-track countries was involving multiple sectors (including health and non-health sectors), mobilizing community and partners, the use of evidence-based decision making, and establishing guiding principles for the overall activities [94]. Therefore, the Ethiopian Federal Ministry of Health (FMOH) can use the experience of such fast-track countries to reduce adolescent pregnancy and prevent its adverse neonatal outcomes, and thereby improve the maternal and child health status in the country.

A non-significant difference in the Apgar score of newborn babies of adolescent and adult women at birth and five minutes after birth was observed. This finding is consistent with previous studies conducted in Cameroon [22], Nepal [95], and USA [2]. However, studies conducted in Thailand [3] and Addis Ababa, Ethiopia [27] showed a higher risk of low Apgar score at one minute after birth for babies born from adolescents compared to adult women. This could be attributed to the difference in the sociodemographic, obstetric, nutritional factors, and study period between the current and previous studies.

This study also found a non-significant difference in the neonatal admission rate to ICU between babies of adolescent and adult women. The finding is different from the previous study conducted in USA [2] that showed a higher risk of neonatal admission among younger adolescent women. In addition, a study conducted in Thailand [74] showed a higher risk of neonatal admission rate among babies of adolescent compared to adult women. The difference could be attributed to sociodemographic, obstetric, nutrition and access to adolescent related health services. In addition, the non-significant difference in the level of low Apgar score at first- and fifth-minute and neonatal admission rate in this study could be related to the low sample size of the study to detect such small differences for these adverse outcomes. Therefore, future larger scale studies that can clearly elucidate the association of Apgar score and adolescent pregnancy in Ethiopia are recommended.

The WHO recommends prevention of adolescent pregnancy as the main strategy to reduce neonatal mortality, especially in developing countries [92]. Interventions like prevention of early marriage, improving the knowledge of adolescents towards SRH issues and the use of contraceptive methods are effective in reducing adolescent pregnancy and related complications [92]. Therefore, the FMOH should strengthen the available programs in prevention of adolescent pregnancy. The use of such interventions can reduce unwanted pregnancy among adolescents, LBW and preterm birth complications, and reduces overall child morbidity and mortality in the country [92].

This study is not without limitations. It was conducted in a hospital setting, and were unable to assess the outcomes of women who gave birth at home, although available report in the study area showed 90% institutional delivery rate [30]. The significant difference for some of the obstetric characteristics (like: proportion of previous pregnancy, mean number of pregnancies, etc.) among adolescent and adult women maybe an artefact of the study design, since this study compared adolescents and adult women. Adolescents are young people and are more likely that the pregnancy was their first, and therefore, the mean number of previous pregnancies will be different. In addition, this study could be underpowered for some of the outcomes. For example, the non-significant difference in the low Apgar score at first and five minutes after birth and neonatal admission to ICU between adolescent and adult women could be because of small sample size for these specific outcomes. In addition, outcomes like stillbirth and neonatal infections were not assessed in this study.

### Conclusions

Adolescent women were less likely to receive antenatal care follow-ups, have a lower mean age at first pregnancy, and less likely to receive iron and folic acid supplementation during pregnancy than adult women. After adjusting for confounding variables, this study found a higher odd of adverse neonatal outcomes among babies born from adolescents compared to adult women. The common forms of adverse neonatal outcomes associated with adolescent pregnancy were LBW and preterm birth. In addition, higher percentage of babies born from adolescents had two or more adverse neonatal outcomes compared to babies of adult women. Addressing the sexual and reproductive health needs of adolescent girls is central to reduce the high rate of child morbidity and mortality in Ethiopia. Prevention of adolescent pregnancy using school and community-based sexuality education and family planning information and service provision programs should be the main focus of health care planners. Moreover, future large-scale studies which can clearly elucidate the association of adolescent pregnancy with neonatal ICU admission and Apgar score level of newborn babies is recommended.

## Supporting information

S1 File Data collection questionnaire in English and Amharic languages. (DOCX)

S2 File Relevant data of this study. (XLSX)
