# Supplementary material for: Adverse neonatal outcomes of adolescent pregnancy in Northwest Ethiopia
Source: PLoS One. 2019 Jun 13;14(6):e0218259. doi: 10.1371/journal.pone.0218259 (PMC6564016; doi:10.1371/journal.pone.0218259)
Supplement: S1 File — (DOCX) [file pone.0218259.s001.docx]

**Questionnaire No./CODE**

| DATE OF INTERVIEW: | Time Interview Started: Hour: _____ Minute: _____  Time Interview Ended: Hour**: _____** Minute: _____ |
| --- | --- |
| INTERVIEWER NAME: |  |
| NAME OF HEALTH FACILITY: |  |

| **Part 1. Address** | | |
| --- | --- | --- |
|  | What is the name of the district you are living? | District_____________ |
|  | What is the urban/rural category of your kebele | 1. Urban 2. Rural |
|  | What is the name of your Kebele and local area? | 1. Kebele__________________ 2. Local area __________________ |
|  | Code for name of the mother |  |

| **Part 2. Socio-demographic characteristics** | | | | |
| --- | --- | --- | --- | --- |
| **S.No.** | **Variables** | **Response** | Skip to | |
|  | In what month and year were you born?  (write in Ethiopian calendar) | Month………………………  Don’t know month…………  Year …………………  Don’t know year………………… | |  |
|  | How old were you at your last birthday? (Compare and correct 101 and/or 102 if inconsistent) | Age in completed years ………… | |  |
|  | Have you ever attended school? | 1. Yes 2. No | | **206** |
|  | What is the highest level of school you attended? | 1. Primary (grade 1-8) 2. Secondary (grade 9-12) 3. Technical/vocational 4. Higher | |  |
|  | What is your ethnicity | 1. Amhara 2. Tigray 3. Oromo 4. Others (specify) __________________ | |  |
|  | What is your religion | 1. Orthodox 2. Catholic 3. Protestant 4. Muslim 5. Others (specify) __________________ | |  |
|  | Your father’s education level | 1. Unable to read and write 2. Can read and write 3. Grade1-6 4. Grade 7-12 5. Collage level and above | |  |
|  | Your mother’s education level | 1. Unable to read and write 2. Can read and write 3. Grade1-6 4. Grade 7-12 5. Collage level and above | |  |
|  | Your father’s occupation | 1. Daily laborer 2. Farmer 3. Civil servant 4. Employed in private sector 5. Has private business 6. Others (specify) __________________ | |  |
|  | Your mother’s occupation | 1. Daily laborer 2. Farmer 3. Civil servant 4. Employed in private sector 5. Has private business 6. Others (specify) __________________ | |  |
|  | What is your usual occupation, that is, what kinds of work do you mainly do? | 1. In school 2. Working, (specify your work) _____________ 3. Neither in school or working 4. Other(specify) __________________ | |  |
|  | Are you currently married or living together with a man as if married? | 1. Currently married 2. Yes, living with a man 3. No, not in union | | 216  216 |
|  | If your answer to question 212 is no, have you ever been married or lived together with a man as if married? | 1. Yes, formerly married 2. Yes, lived with a man 3. No | |  |
|  | If your answer to question 213 is no, what is your marital status now: are you widowed, divorced, or separated? | 1. Widowed 2. Divorced 3. Separated | |  |
|  | Is your husband/partner living with you now or is he staying elsewhere? | 1. Living with me 2. Staying elsewhere | |  |
|  | If married, your husband’s occupation | 1. Daily laborer 2. Farmer 3. Civil servant 4. Employed in private sector 5. Has private business 6. Others (specify) __________________ | |  |
|  | Living arrangement, with whom are you living now? | 1. With husband/spouse 2. Father and mother 3. With relatives 4. Friends 5. Alone 6. Other, specify ___________________ | |  |

| Part 3. Questions related to household assets | | | |
| --- | --- | --- | --- |
| **S.No.** | **Variables** | **Response** | Skip to |
|  | What is the main material of the floor? | 1. Earth/sand 2. Dung 3. Wood planks 4. Palm/bamboo 5. Ceramic tiles 6. Cement 7. Carpet 8. Other (specify)________________________ |  |
|  | Main roof material of your house | 1. Corrugated iron 2. Thatch 3. Cement/concrete 4. Plastic sheet 5. Other (specify)________________________ |  |
|  | What is the main source of drinking water for  members of your household? | 1. Piped water 2. Water from well 3. Surface water 4. Other (specify)______________________ |  |
|  | What kind of toilet facilities do you have in  your home? | 1. Flush toilet 2. Pit toilet/latrine 3. No facility/bush/field 4. Other (specify)______________________ |  |
|  | What type of fuel do you mainly use for  cooking in your household? | 1. Electricity 2. Firewood, straw 3. Charcoal 4. Biogas 5. Kerosene 6. Other (specify)______________________ |  |
|  | Does your household have electricity? | 1. Yes 2. No |  |
|  | Own the house living in? | 1. Yes 2. No |  |
|  | Does your household own:   1. A landline telephone? 2. A refrigerator? 3. A radio that is in working order? 4. A television that is in working order? 5. A bed with cotton/sponge/spring mattress? 6. Farm land 7. Have cattle   How many cattle?   1. Have horse/ donkey   How many?   1. Have sheep/goats?   How many? | **YES NO**  ............1.......... 2  ............1.......... 2  ............1.......... 2  ............1.......... 2  ............1.......... 2  ............1.......... 2  ............1.......... 2  ………………..  ............1.......... 2  ………………..  ............1.......... 2  ……………….. |  |
|  | Does any member of your household own:   1. A cell phone? 2. A bicycle? 3. A motorcycle or motor scooter? 4. A car or truck? 5. An animal-drawn cart? | **YES NO**  ............1.......... 2  ............1.......... 2  ............1.......... 2  ............1.......... 2  ............1.......... 2 |  |
|  | How many rooms in your house are used for sleeping? | __________________ (write in number) |  |
|  | Total number of family members living in your household? | ________________ (write in number) |  |
|  | How much is your household monthly income (write in Ethiopian birr (ETB)) | _______________ ETB |  |

| **Part 4. Past obstetric history of respondents** | | | |
| --- | --- | --- | --- |
| **S.No** | **Variables** | **Response** | **Skip to** |
|  | What was the age at which you married for the first time? | ________________ years old |  |
|  | Have you ever been pregnant, other than this pregnancy | 1. Yes, _________ times 2. No | 410 |
|  | What was your age at your first pregnancy | ________ years old |  |
|  | Have you ever aborted? | 1. Yes, _______ times 2. No |  |
|  | Have you given birth to a child? | 1. Yes, ______ children 2. No |  |
|  | How many live births did you have? | ___________ (write in number) |  |
|  | Did you have stillbirths? | 1. Yes, _________ times 2. No |  |
|  | Have you experienced an infant death? | 1. Yes, _________ times 2. No |  |
|  | How many home deliveries did you have | __________(write in number) |  |
|  | How many institutional deliveries did you have | __________(write in number) |  |
|  | Thinking back to just before you got pregnant for the current pregnancy, how did you feel about becoming pregnant? | 1. I wanted to be pregnant sooner 2. I wanted to be pregnant later 3. I wanted to be pregnant then 4. I didn’t want to be pregnant then or at any time in the future |  |
|  | When you got pregnant with your current pregnancy, were you or your husband or partner doing anything to keep from getting pregnant? (ex. using birth control methods.) | 1. Yes 2. No | 414 |
|  | If yes to question number 211, Which of the following methods were you/your husband using before you got pregnant? | 1. Pills 2. Injectables 3. IUCD 4. Implants 5. Condoms 6. Rythum method 7. Other (specify) ___________________ |  |
|  | (Now I have some questions about the future.) After the child you are expecting now, would you like to have another child, or would you prefer not to have any more children? | 1. Have a/another children 2. No more 3. Undecided/don’t know 4. Other (specify) __________________ |  |
|  | After the child you are expecting now, how long would you like to wait before the birth of another child? | 1. Years _____________________ 2. Months ____________________ 3. Soon/now 4. Don’t know |  |
|  | Did you attend pregnancy checkups/ANC for the current pregnancy? | 1. Yes, _________ times 2. No | 420 |
|  | How many weeks or months pregnant were you when you first received antenatal care for the pregnancy? | Number of months \|____\| \|____\|  Weeks ______ |  |
|  | How many times did you receive antenatal care during the current pregnancy? | Number \|____\| \|____\|  Don’t know--------------------- 99 |  |
|  | Where did you receive antenatal care for the current pregnancy? | 1. Hospital 2. Health center 3. Private hospital/clinic 4. Other (Specify) _________________ |  |
|  | During this pregnancy, were you given an injection in the arm to prevent the baby from getting tetanus, that is, convulsions after birth? | - - - 1. Yes       2. No |  |
|  | During this pregnancy, how many times did you receive tetanus injection? | _____________ times |  |
|  | Have you received Iron and folic acid supplementation? | 1. Yes, for _________ months duration 2. No |  |
|  | How far is it from your residential home to the nearby health facility (health center, hospital)? | Distance in kms_________ Do not know.........................99 |  |
|  | How long does it take to walk from here to the nearby health facility (health center, hospital)? | Minutes.................................. Don’t know minutes....................99 |  |
|  | Previous history of hypertension | 1. Yes 2. No |  |
|  | Family history of hypertension | 1. Yes 2. No |  |
|  | Family history of diabetes mellitus | 1. Yes 2. No |  |
|  | Do you have any other family related health condition | 1. Yes, specify _____________________ 2. No |  |
|  | Did you had malaria attack during the current pregnancy? | 1. Yes 2. No |  |
|  | Did you have any of the following problems during the current pregnancy?   1. Vaginal bleeding 2. Severe headache 3. Severe nausea, vomiting, or dehydration 4. High blood pressure, hypertension 5. Fever 6. Abdominal pain 7. Other _______________________ | **Yes No**   1. 2 2. 2 3. 2 4. 2 5. 2 6. 2 7. 2 |  |
|  | What have you done to alleviate the problem you had? | 1. I was not sick 2. Nothing 3. Treated by health professional 4. Took traditional medicine 5. Other, specify ________________ |  |
|  | During any of your prenatal care visits did a doctor, nurse, or other health care worker talk with you about any of the things listed below?   1. How drinking alcohol or smoking cigarettes during pregnancy could affect your baby 2. The signs and symptoms of preterm labor (labor more than 3 weeks before the baby is due) 3. Signs of pregnancy Complications? 4. What to do if your labor starts early 5. What to do for Pregnancy complication 6. Getting tested for HIV (the virus that causes AIDS) 7. Where to go for delivery 8. Post-natal period (for instance breastfeeding, nutrition, care for the child)? 9. Possible mood changes in the postnatal period? | **Yes No**   1. 2 2. 2 3. 2 4. 2 5. 2 6. 2 7. 2 8. 2 9. 2 |  |
|  | If you didn’t attend, what was the reason? | 1. I don’t know there is such a service 2. The health unit is far away from home 3. I don’t have any health problem 4. I don’t like the way the health professionals handle clients 5. My belief doesn’t allow me 6. Other, specify ______________ |  |
|  | During your current pregnancy, did the healthcare providers told you to bring your spouse's to prenatal care? | - - - 1. Yes       2. No |  |
|  | Did your spouse/partner came to health facility for antenatal care purpose during the current pregnancy? | 1. Yes 2. No |  |
|  | At any time during your current pregnancy, did your husband/partner push, hit, slap, kick, choke, or physically hurt you in any other way? | - - - 1. 1. Yes       2. 2. No |  |
|  | At any time during your current pregnancy, did your husband/partner physically forced to do sexual intercourse, had sexual intercourse when you do not want? | - - - 1. Yes       2. No |  |
|  | At any time during your current pregnancy, did your husband/partner insulted, Humiliated, done something to scare you, or threatened to hurt you? | 1. Yes 2. No |  |
|  | History of hospital admission during current pregnancy | 1. Yes 2. No |  |
|  | If yes to question 334, what was the reason for hospital/health center admission? | _____________________________ |  |
|  | Were you admitted to a maternity waiting homes during the current pregnancy before childbirth? | 1. Yes 2. No |  |
|  | If your answer to question number 440 is yes, for how many days were you admitted to maternity waiting homes before childbirth? | ________ days  ________ hours |  |

| **Part 5. Questions related to substance abuse** | | | | | | | | |  |
| --- | --- | --- | --- | --- | --- | --- | --- | --- | --- |
| Now I am going to ask you some questions about Substance Use | | | | | | | | |  |
| S.No. | Question | | | Response | | | | | Skip |
|  | Did you ever drink alcoholic beverages? Like (Tela, Teji, areki or Beer). | | | 1. Never 2. Drink daily 3. Once or twice a week 4. Others (specify)_______ 5. No response | | | | | 503 |
|  | If yes to Qn 401, did you drink alcoholic beverages during the current pregnancy? Like (Tela, Teji, areki or Beer). | | | 1. Yes 2. No | | | | |  |
|  | Did you ever smoke tobacco products, such as cigarettes? | | | 1. Never 2. Smoke daily 3. Once or twice a week 4. Others (specify)_______ 5. No response | | | | | 505 |
|  | If yes to Qn 403, Did you smoke tobacco products, such as cigarettes during the current pregnancy? | | | 1. Yes 2. No | | | | |  |
|  | Have you ever chew *Khat*? | | | 1. Never 2. Chew daily 3. Once or twice a week 4. Others (specify)_______ 5. No response | | | | | 507 |
|  | If yes to Qn 405, did you chew *Khat* during the current pregnancy? | | | - - - 1. Yes       2. No | | | | |  |
|  | Have you ever used other drugs such as hashish/Shisha? | | | 1. Never 2. Use daily 3. Once or twice a week 4. Others (specify)_______ 5. No response | | | | | 601 |
|  | If yes to Qn 407, did you use these (hashish/Shisha) during the current pregnancy? | | | 1. Yes 2. No | | | | |  |
| **Part 6. Maternal outcomes during labor and delivery** | | | | | | | | | |
| **S.No** | | **Variables** | **Response** | | | | | | Skip to |
|  | | Is the mother delivered at this health center or referred to higher health facility? | 1. Delivered at this facility 2. Referred to higher health facility | | | | | | 604 |
|  | | If the mother is referred what is the name of the health facility the mother is referred to? | ____________________________ | | | | | |  |
|  | | If the mother is referred to higher health facility, what was the indication for referral? (more than one answer is possible) | 1. Prolonged labour 2. Fetal distress 3. Previous cesarean section scar 4. Preclapsia / eclampsia 5. Previous fistula history 6. Other(specify):   _______________ | | | | | |  |
|  | | What was the delivery type/mode of delivery?  (More than one option is possible) | 1. Spontaneous vaginal delivery/normal birth 2. Caesarean Section 3. Forceps delivery 4. Vacuum extraction 5. Other procedures _________________ | | | | | |  |
|  | | Did the woman undergo Episiotomy? | - - - 1. Yes       2. No | | | | | |  |
|  | | If delivery was by Caesarean Section, what was the reason for operative delivery  (More than one response is possible) | 1. Breech presentation 2. Failure to progress in labor 3. Failure to deliver 4. Fetal malposition 5. Non-reassuring fetal heart rate 6. Placenta previa 7. Other, specify _______________ | | | | | |  |
|  | | If delivery was by Caesarean Section (C/S), what was the reason the type of caesarean section? | - - - 1. Lower uterine segment transverse C/S       2. Classical C/S       3. Inverted T C/S       4. Other, specify _______________ | | | | | |  |
|  | | What was the presentation of the fetus during delivery? | 1. Cephalic prsentation 2. Breech presentation 3. Shoulder presentation 4. Face presentation 5. Other(specify) ___________ | | | | | |  |
|  | | What was the position of the fetus during delivery? | 1. Ocipito anterior position 2. Ocipito posterior position 3. Right ocipito lateral position 4. Left ocipito lateral position 5. Other (specify) _____________ | | | | | |  |
|  | | Gestational age of the pregnant mother during labor/delivery? | \|____\| \|____\| weeks of gestation | | | | | |  |
|  | | Time duration from initiation of labor to full cervical dilatation | ________ hours | | | | | |  |
|  | | Did the mother undergo obstetric Induction of labour? | 1. Yes 2. No | | | | | |  |
|  | | Did the mother had premature rupture of membrane (PROM)? | 1. Yes 2. No | | | | | |  |
|  | | Who assisted the delivery?  (more than one answer is possible) | 1. Doctor 2. Midwife 3. Nurse 4. Health officer 5. Obstetrics and gynecology specialist 6. Other, specify ___________ | | | | | |  |
|  | | Does the mother have any of the following complications?   1. Preeclampsia ………………………………………………………… 2. Eclampsia ……………………………………………………………… 3. Anteparum hemorrhage (APH) ……………………………… 4. Postpartum hemorrgae (PPH) ………………………………… 5. Shock…………………………………………………………………………… 6. Other obstetric complications (specify)___________.. | **Yes (1) No (2)**  ……. 1 …. 2  ……. 1 …. 2  ……. 1 …. 2  ……. 1 …. 2  ……. 1 …. 2  ……. 1 …. 2 | | | | | |  |
|  | | If the woman has preeclampsia/eclampsia, was there proteinuria (protein in the urine)? | 1. Proteinuria present 2. Proteinuria absent | | | | | | **618** |
|  | | If protein urea present, what was the urine protein measurement? (write the lab result in the space provided) | **_____________________** | | | | | |  |
|  | | What is the hemoglobin level of the mother during labor and delivery? | _____________ (write the result in g/dl) | | | | | |  |
|  | | Maternal preventive services   1. Vitamin A given to the mother…………………………... 2. HIV testing accepted ……………………………………… 3. HIV test result……………………………………………… | **Yes (1) No (2)**  ……. 1 …. 2  ……. 1 …. 2.  Positive 2. Negative | | | | | |  |
|  | | What is the maternal status after delivery? | 1. Stable 2. Unstable/ deteriorated 3. Died | | | | | | **621** |
|  | | If the mother is not alive, what was the main cause of death diagnosed? | 1. Post-partum hemorrhage (PPH) 2. Hypertension disorder (Specify): ___________ 3. Infectious disease diagnosis (Specify): _______ 4. Other(Specify)__________________ | | | | | |  |
|  | | | | | | | | | |
| **Part 7. Newborn Outcomes** | | | | | | | | | |
|  | | What is the sex of the newborn baby? | | | 1. Male 2. Female | | | |  |
|  | | What was the newborn outcome | | | 1. Alive 2. Dead | | | | 717 |
|  | | What was the birthweight of the baby (in grams)? | | | ____________________ grams | | | |  |
|  | | APGAR score 1^st^ minute after birth | | | __________________ (write the score) | | | |  |
|  | | APGAR score 5^th^ minute after birth | | | __________________ (write the score) | | | |  |
|  | | New born preventive services   1. BCG given to the newborn…………………………....... 2. OPV 0 given to the newborn………………………….... | | | **Yes (1) No (2)**  ……. 1 …. 2  ……. 1 …. 2. | | | |  |
|  | | Any evidence of jaundice in the new born baby in first 24 hours of life, eg. yellow palms and soles at any age. | | | 1. Yes 2. No | | | |  |
|  | | Does the newborn has any form of congenital malformation? | | | 1. Yes 2. No | | | | 710 |
|  | | If the newborn has congenital malformation, what was the type/diagnosis of malformation? | | | **___________________________________** | | | |  |
|  | | Does the newborn baby have any of the following problems?   1. Prematurity ……………………………………………………………………. 2. Sepsis………………………………………………………………………………. 3. Respiratory distress ………………………………………………………... 4. Perinatal asphyxia………………………………………………………….. 5. Anemia…………………………………………………………………………… 6. Congenital malformation ……………………………………………….. 7. Meconium aspiration …………………………………………………….. 8. Other (specify) ___________________........................... | | | | **Yes (1) No (2)**  ……. 1 …. 2  ……. 1 …. 2  ……. 1 …. 2  ……. 1 …. 2  ……. 1 …. 2  ……. 1 …. 2  ……. 1 …. 2  ……. 1 …. 2 | | |  |
|  | | Treatment given to the newborn baby   1. Oxygen/ resuscitation…………………………………………. 2. Kangaroo mother care (KMC) …………………….. 3. Antibiotics …………………………………………………………… 4. Glucose………………………………………………………………… 5. Blood transfusion………………………………………………….. 6. Other (specify) ___________________....................... | **Yes (1) No (2)**  ……. 1 …. 2  ……. 1 …. 2  ……. 1 …. 2  ……. 1 …. 2  ……. 1 …. 2  ……. 1 …. 2 | | | | | |  |
|  | | If treatment is given to the newborn baby, what was the treatment outcome?   1. Improved……………………………………………………………… 2. No change……………………………………………………………. 3. Died……………………………………………………………………… 4. Referred……………………………………………………………….. | **Yes (1) No (2)**  ……. 1 …. 2  ……. 1 …. 2  ……. 1 …. 2  ……. 1 …. 2 | | | | | |  |
|  | | Did the mother identified on the type of breast feeding? | 1. Yes 2. Not at all | | | | | |  |
|  | | If mother starts breast feeding, within how many hours after childbirth did she start breastfeeding? | ____________ hours after childbirth | | | | | |  |
|  | | Was the newborn admitted to neonatal intensive care unit (ICU)? | 1. Yes 2. No | | | | | |  |
|  | | If yes to question 615, what was the reason for the neonatal ICU admission? | 1. Prematurity 2. Infection 3. Asphyxia 4. Congenital malformation 5. Other (specify) ___________________ | | | | | |  |
|  | | If the baby died, what was the cause of death?   1. Prematurity ………………………………………………………………… 2. Infection …………………………………………………………………….. 3. Asphyxia ……………………………………………………………………. 4. Congenital malformation …………………………………………… 5. Other (specify) ___________________....................... | **Yes (1) No (2)**  ……. 1 …. 2  ……. 1 …. 2  ……. 1 …. 2  ……. 1 …. 2  ……. 1 …. 2 | | | | | |  |
|  | | If dead, what was the type? | 1. Still birth 2. Death of the baby in the health facility after live birth | | | | | |  |
|  | | | | | | | | | |
| **Part 8. Data collection before discharge from the health facility** | | | | | | | | | |
| **S.No** | | **Variables** | | | | | **Response** | | Skip to |
|  | | Does the mother has Signs and symptoms of post partum hemorrhage?   1. Sudden and profuse blood loss or persistent increase blood loss.............. 2. Faintness……………………………………………………………………………… 3. Dizziness ……………………………………………………………………………………….. 4. Palpitations/tachycardia…………………………………………………………………… 5. Other (specify) ___________________........................................ | | | | | **Yes (1)** **No (2)**  ……. 1 …. 2  ……. 1 …. 2  ……. 1 …. 2  ……. 1 …. 2  ……. 1 …. 2 | |  |
|  | | Signs and symptoms of pre-eclampsia/eclampsia (after birth)?   1. Headaches………………………………………………………………………… 2. Visual disturbances………………………………………………………….. 3. Nausea………………………………………………………………………………. 4. Vomiting…………………………………………………………………………... 5. Epigastric or hypochondrial pain…………………………………….. 6. Feeling faint……………………………………………………………………… 7. Convulsions……………………………………………………………………… 8. Other (specify) ________________...................... | | | | | **Yes (1**) **No (2)**  ……. 1 …. 2  ……. 1 …. 2  ……. 1 …. 2  ……. 1 …. 2  ……. 1 …. 2  ……. 1 …. 2  ……. 1 …. 2  ……. 1 …. 2 | |  |
|  | | Does the mother have any signs and symptoms of infection?   1. Fever…………………………………………………………………………………. 2. Shivering…………………………………………………………………………… 3. Abdominal pain………………………………………………………………… 4. Offensive vaginal discharge.…………………………………………… 5. Other (specify) ________________................................ | | | | | **Yes (1)** **No (2)**  ……. 1 …. 2  ……. 1 …. 2  ……. 1 …. 2  ……. 1 …. 2  ……. 1 …. 2 | |  |
|  | | Duration of stay in health facility after birth before discharge to home? (write the answer in hours) | | | | | | _____ hours |  |

**Questionnaire No./CODE**

**RH-**

| **ክፍል 1. የተጠያቂዋ አድራሻ** | | |
| --- | --- | --- |
|  | የምትኖሪበት የወረዳ ስም ማን ይባላል? | ወረዳ ስም _____________ |
|  | የምትኖሪበት ቀበሌ የከተማ / የገጠር ክፍል ነው? | 1. ከተማ 2. ገጠር |
|  | የምትኖሪበት የቀበሌ እና የጎጥ ስም ማን ይባላል? | 1. ቀበሌ _________________ 2. ጎጥ_____________________________ |
|  | የእናት ስም ኮድ |  |

| DATE OF INTERVIEW: | Time Interview Started: Hour: _____ Minute: _____  Time Interview Ended: Hour**: _____** Minute: _____ |
| --- | --- |
| INTERVIEWER NAME: |  |
| NAME OF HEALTH FACILITY: |  |

| **ክፍል 2. የተጠያቂ ማህበራዊ ባህሪያት በተመለከተ የሚጠየቁ ጥያቄዎች** | | | | |
| --- | --- | --- | --- | --- |
| **ተ.ቁ** | **ጥያቄዎች** | | **መልስ** | Skip to |
|  | በየትኛው ወር እና ዓመት ተወለዱ?  (በኢትዮጵያ የቀን መቁጠሪያ/ ዓመተ ምህረት ይጻፉ) | | ወር…………………………>>  ወሩን አላውቀውም…………  ዓመት ……………………>>  ዓመቱን አላውቀውም………………… |  |
|  | ባለፈው የልደት ቀንዎ እድሜዎት ምን ያህል ነበር?  (ጥያቄ 201 እና 202 ካልተመሳሰለ አስተካክል/ድጋሚ ጠይቅ/ቂ) | | ዕድሜ ………… ዓመት |  |
|  | ትምህርት ተከታትለዋል? | | 1. አዎ 2. የለም | 206 |
|  | እርስዎ የተማሩበት ከፍተኛው የትምህርት ቤት ደረጃ ምን ያህል ነው? | | 1. አንደኛ ደረጃ (ከ1-8ኛ ክፍል 2. ሁለተኛ ደረጃ (ከ9-12ኛ ክፍል) 3. ቴክኒክ እና ሞያ 4. ከፍተኛ ትምህርት |  |
|  | የእርስዎ ጎሳ /ብሄር ምንድነው | | 1. አማራ 2. ትግራይ 3. ኦሮሞ 4. ሌሎች (ይግለጹ)_________ |  |
|  | ሃይማኖትሽ ምንድን ነው | | - - - 1. ኦርቶዶክስ       2. ካቶሊክ       3. ፕሮቴስታንት       4. ሙስሊም       5. ሌሎቹ (ይግለጹ) __________________ |  |
|  | የአባትሽ የትምህርት ደረጃ | | 1. ማንበብና መጻፍ አይችልም 2. ማንበብና መጻፍ ይችላል 3. ከ 1-6 ክፍል 4. ከ 7 ኛ እስከ 12 ኛ 5. የኮሌጅ ደረጃ እና በላይ |  |
|  | የእናትሽ የትምህርት ደረጃ | | 1. ማንበብና መጻፍ አትችልም 2. ማንበብና መጻፍ ትችላለች 3. ከ 1-6 ክፍል 4. ከ 7 ኛ እስከ 12 ኛ 5. የኮሌጅ ደረጃ እና በላይ |  |
|  | የአባትሽ ሥራ | | - - - 1. የቀን ሰራተኛ       2. ገበሬ       3. የመንግስት ሰራተኛ       4. በግሉ ዘርፍ ውስጥ ተቀጥሮ ይሠራል       5. የግል ንግድ አለው       6. ሌሎች (ይግለጹ)________ |  |
|  | የእናትሽ ሥራ | | - - - 1. የቀን ሰራተኛ       2. ገበሬ       3. የመንግስት ሰራተኛ       4. በግሉ ዘርፍ ውስጥ ተቀጥራ ትሠራለች       5. የግል ንግድ አለው  1. ሌሎች (ይግለጹ)____________ |  |
|  | የተለመደው ሥራሽ ምንድን ነው? (ምን ዓይነት ሥራዎችን በዋነኝነት ያከናውናሉ?) | | 1. በትምህርት ቤት 2. በስራ (ስራዎን ይግለጹ) __________ 3. በትምህርት ቤት ወይም በሥራ ላይ አይደለም 4. ሌላ (ይግለጹ) _____________ |  |
|  | በአሁኑ ጊዜ ባለትዳር ነሽ?  (ካልሆንሽ ከጋብቻ ውጭ ከወንዶች ጋር እየኖርሽ ነው?) | | 1. በአሁኑ ጊዜ የተጋባሁ ነኝ 2. ትዳር አልያዝኩም፣ ነገር ግን ከወንድ ጋር እየኖርሁ ነው 3. አልተጋባሁ፣ ብቻዬን ነው የምኖረው | 216  216 |
|  | ለጥያቄ 212 መልስዎ አልተጋባሁም ከሆነ, ካሁን በፊት ትዳር ይዘው ወይም ከወንድ ጋር አብረው ኖረው ያውቃሉ? | | - - - 1. አዎ, ቀደም ሲል አግብቻለሁ       2. አዎ ከወንድ/ዶች ጋር እኖር ነበር       3. አይ፣ አግብቼ አላውቅም ወይንም ከወንድ ጋር አብሬ አልኖርኩም |  |
|  | ለጥያቄ ቁጥር 213 መልስዎ “አይ አግብቼ አላውቅም” ከሆነ የጋብቻ ሁኔታዎ አሁን ምን ይመስላል? | | - - - 1. ባለቤቴ ሞቶብኛል       2. የተፋታሁ ነኝ       3. የተለያየሁ ነኝ |  |
|  | ባለቤትሽ ወይም ጓደኛሽ አሁን ከአንቺ ጋር ይኖራል? ወይስ ሌላ ቦታ ነው ያለው? | 1. ከእኔ ጋር ነው የሚኖረው 2. 2. ሌላ ቦታ ነው የሚኖረው | |  |
|  | ካገባሽ፣ የባለቤትሽ ሥራ ምንድን ነው? | | - - - 1. የቀን ሰራተኛ       2. ገበሬ       3. የመንግስት ሰራተኛ       4. በግሉ ዘርፍ ውስጥ ተቀጥሬ እሠራለሁ       5. የግል ንግድ አለኝ       6. ሌሎች (ይግለጹ)______________ |  |
|  | አሁን ከማን ጋር ነው የምትኖሪው? | | - - - 1. ከባለቤት/ ከትዳር ጓደኛ ጋር       2. አባትና እናቶች       3. ከዘመዶች       4. ጓደኞች       5. ብቻዬን       6. ሌላ, (ይግለጹ)___________ |  |

| **ክፍል 3. ስለመኖርያ ቤት/ቤተሰብ የሚመለከቱ መጠይቆች፣** አሁን ስለቤተሰብዎ እና ስለሚኖሩበት ቤት አንዳንድ ጥያቄዎችን እጠይቃለሁ፡፡ | | | | | | |
| --- | --- | --- | --- | --- | --- | --- |
| ተ.ቁ | ጥያቄዎች | መልስ | | | Skip to | |
|  | የምትኖሩበት ቤት ወለል ከምንድን ነው የተሰራው? | 1. ከአፈር/አሸዋ 2. በእበት የተለቀለቀ 3. ከእንጨት 4. ከሸንበቆ/ቀርቅሃ 5. ከሴራሚክ 6. ከሲሚንቶ 7. ከምንጣፍ 8. ሌላ ካለ (ይገለፅ)...................... | | | |  |
|  | የመኖርያ ቤትዎ ጣሪያ ከምንድን ነው የተሰራው? | - - - 1. በቆርቆሮ የተሰራ       2. የሳር ቤት       3. ከሲሚንቶ       4. ከፕላስቲክ የተሰራ ጣርያ       5. ሌላ(ይገለፅ) .................................... | | | |  |
|  | ለቤተሰብ አባላት የሚሆን ዋና የመጠጥ ውኃ ምንጭ ምንድን ነው? | የቧንቧ ውሃ  ከጥልቅ ጉድጓድ የተገኘ ውሃ  የምንጭ/የወንዝ ውሃ  ሌላ ካለ (ይገለፅ) .................................... | | | |  |
|  | በቤትዎ ውስጥ ምን ዓይነት መጸዳጃ ቤት/ቶች አሉዎት? | 1. በውሃ የሚሰራ ሽንት ቤት 2. የጉድጓድ ደረቅ ሽንት ቤት 3. ሽንት ቤት የለም/መስክ ላይ /ጫካ ላይ 4. ሌላ ካለ (ይገለፅ)_________________________ | | | |  |
|  | በቤተሰብዎ ውስጥ ምግብ ለማብስል የምትጠቀሙበት ነዳጅ ምንድን ነው ? | 1. የኤሌክትሪክ ኃይል 2. እንጨት/ ገለባ 3. ከሰል 4. ባዮጋዝ 5. ነጭ ጋዝ 6. ሌላ ካለ (ይገለፅ) ...................................... | | | |  |
|  | የእርስዎ ቤተሰብ/መኖሪያ ቤት የኤሌክትሪክ ኃይል አለው? | 1. አዎ 2. የለም | | | |  |
|  | በአሁኑ ሰዓት የሚኖሩበት ቤት የራስዎ/ነው? | 1. አዎ 2. የለም | | | |  |
|  | በእርስዎ ቤተሰብ/መኖሪያ ቤት የትኞቹ የቤት እቃዎች አሉ?   1. መደበኛ/ተንቀሳቃሽ ያልሆነ/የቤት ስልክ……………………… 2. ማቀዝቀዣ/ፍሪጅ……………………………………………………… 3. ሬዲዮ……………………………………………………………… 4. ቴሌቪዥን…………………………………………………………… 5. ከጥጥ / ስፖንጅ / የተሰራ ፍራሽ ያለው አልጋ?................... 6. የእርሻ መሬት አላችሁ…………………………………………… 7. ከብቶች አላችሁ…………………………………………………….   ምን ያህል ከብቶች አላችሁ?.............................................   1. ፈረስ / አህያ…………………………………………………………   ምን ያህል ፈረስ / አህያ አላችሁ?.......................................   1. በግ / ፍየል አላችሁ?........................................................   ምን ያህል በግ / ፍየል አላችሁ?......................................... | አዎ የለም  ............1.......... 2  ............1.......... 2  ............1.......... 2  ............1.......... 2  ............1.......... 2  ............1.......... 2  ............1.......... 2  …………..  ............1.......... 2  ..…………  ............1.......... 2  ………….. | | | |  |
|  | ከቤተሰብዎ መካከል የሚከተሉት እቃዎች ያለው የቤተሰብ አባል አለ ?   1. የሞባይል ስልክ?……………………………………………………………. 2. ብስክሌት?…………………………………………………………………… 3. ሞተርሳይክል ወይም ሞተር……………………………………………… 4. መኪና?………………………………………………………………………… 5. በእንስሳት የሚሳብ ጋሪ?………………………………………………. | | አዎ የለም  ............1.......... 2  ............1.......... 2  ............1.......... 2  ............1.......... 2  ............1.......... 2 | | |  |
|  | በቤታችሁ ውስጥ ለመተኛት/ለመኝታ ክፍል የሚሆን ምን ያህል ክፍሎች ይጠቀማሉ? | | | ______________የመኝታ ክፍሎች (በቁጥር ይገለፅ) | |  |
|  | በቤትዎ ውስጥ የሚኖሩ የቤተሰብዎ ጠቅላላ ቁጥር ስንት ነው? | | | ________________ (በቁጥር ይገለፅ) | |  |
|  | የእርስዎ ቤተሰብ ወርሃዊ ገቢ ምን ያህል ነው? (በኢትዮጲያ ብር ይገለፅ ) | | | _______________ ብር | |  |

| **ክፍል 4. ስለ ፅንስ እና የወሊድ ታሪክን በተመለከተ** | | | | | | | | | | | |
| --- | --- | --- | --- | --- | --- | --- | --- | --- | --- | --- | --- |
| **ተ.ቁ** | **ጥያቄዎች** | | | **መልስ** | | | **Skip to** | | | |  |
|  | ካገባሽ፣ ለመጀመሪያ ግዜ ለጋብቻ ያገባሽበት ዕድሜ ስንት ነበር? | | | ________________ ዓመት | | | | |  | |  |
|  | ከዚህ እርግዝና ሌላ ካሁን በፊት አርግዘሽ ታውቂያለሽ? | | | 1. አዎ, _________ ጊዜ(ቁጥር ይፃፍ) 2. የለም | | | | | 410 | |  |
|  | በመጀመሪያው የእርግዝና ጊዜ ዕድሜሽ ምን ያህል ነበር? | | | _______________ ዓመት | | | | |  | |  |
|  | አስወርዶሽ ያውቃል? | | | 1. አዎ, _________ ጊዜ(ቁጥር ይፃፍ) 2. የለም | | | | |  | |  |
|  | ካሁን በፊት ልጅ ወልደሽ ታውቂያለሽ? | | | 1. አዎ, _________ ልጆች(ቁጥር ይፃፍ) 2. የለም | | | | |  | |  |
|  | ስንት በሂወት የተወለዱ ህፃናት ቁጥር አሉሽ? | | | ___________ (በቁጥር ይፃፉ) | | | | |  | |  |
|  | ካሁን በፊት ሞተው የተወለዱ ህፃናት ነበሩሽ? | | | 1. አዎ, _________(በቁጥር ይፃፉ) 2. የለም | | | | |  | |  |
|  | ካሁን በፊት የሕፃን ሞት አጋጥሞሽ ያውቃል? | | | 1. አዎ, _________(በቁጥር ይፃፉ) 2. የለም | | | | |  | |  |
|  | ካሁን በፊት በቤት ውስጥ/ከህክምና ተቋም ውጭ ልጅ ወልደሽ ታውቂያለሽ? | | | 1. አዎ, _________ጊዜ (በቁጥር ይፃፉ) 2. የለም | | | | |  | |  |
|  | ካሁን በፊት በጤና ተቋም ውስጥ ልጅ ወልደሽ ታውቂያለሽ? | | | 1. አዎ, _________ጊዜ (በቁጥር ይፃፉ) 2. የለም | | | | |  | |  |
|  | የአሁኑን እርግዝና ከመፀነስሽ በፊት፣ ስለእርግዝናው ምን ታስቢ ነበር? | | | - - - 1. በጊዜው ለመፀነስ ፈልጌ ነበር       2. ትንሽ መቆየት እፈልግ ነበር       3. በጊዜው ለመፀነስ አልፈለኩም ነበር፣ አሁን ግን ፈልጌዋለሁ       4. በጊዜው ወይንም ከዚያ በኋላ ለመፀነስ አልፈለኩም ነበር | | | | |  | |  |
|  | በአሁኑ እርግዝና ወቅት፣ አንቺ ወይም ባለቤትሽ እርግዝናን ለመከላከል ሞክራችሁ ነበር? (ለምሳሌ የወሊድ መቆጣጠርያ ዘዴዎችን በመጠቀም) | | 1. አዎ 2. የለም | | | | | | 414 | |  |
|  | ለጥያቄ ቁጥር 412 መልስሽ “አዎ” ከሆነ፣ ከሚከተሉት የእርግዝና ዘዴዎች መካከል የትኛውን ዘዴ ተጠቅመሻል? | | 1. በአፍ የሚወሰዱ የእንክብለ መድሃኒቶች 2. የእርግዝና መከላከያ መርፌ 3. በማህፀን ውስጥ የሚቀበር ሉፕ 4. ክንድ ላይ የሚቀበር መድሃኒት 5. ኮንዶም 6. በተፈጥሯዊ መንገድ 7. ሌላ፣ ይግለጹ ______________ | | | | | | |  |  |
|  | (ስለወደፊቱ አንዳንድ ጥያቄዎች አሉኝ).  አሁን ከምትጠብቂው ልጅ በኋላ ሌላ ልጅ እንዲኖርሽ ትፈልጊያለሽ? ወይስ ተጨማሪ ልጅ ላለመውለድ ትመርጫለሽ? | | | - - - 1. ሌላ ልጅ ወይም ልጆች እፈልጋለሁ       2. የለም ሌላ ልጅ አልፈልግም/ይበቃኛል       3. አልወሰንኩም / አላውቅም       4. ሌላ፣ ይግለጹ ______________ | | | | |  | |  |
|  | አሁን የምትጠብቂውን ልጅ ከወለድሽ በኋላ፣ ሌላ ልጅ ከመወለድሽ በፊት ለምን ያህል ጊዜ መጠበቅ ትፈልጊያለሽ? (በቁጥር ይፃፍ) | | | 1. ______________ዓመት 2. ______________ወራት 3. በቅርቡ / አሁን 4. አላውቅም | | | | |  | |  |
|  | በአሁኑ የእርግዝና ወቅት፣ በጤና ተቋም የእርግዝና ክትትል / አድርገሻል? | | | 1. አዎ 2. ክትትል አላደረኩም | | | | | 420 | |  |
|  | ካሁን በፊት ክትትል አድርገሽ ከሆነ፣ ለመጀመርያ ጊዜ የእርግዝና ክትትል ስታደርጊ፣ የምን ያክል ወራት/ሳምንታት ነብሰ ጡር ነበርሽ? | | | ወራት \|____\| \|____\| አላውቅም ----------------- 99  ሳምንታት **_______________** | | | | |  | |  |
|  | በአሁኑ የእርግዝና ወቅት፣ በጠቅላላው ምን ያክል ጊዜ የጤና ተቋም የእርግዝና ክትትል አድርገሻል? | | | በቁጥር \|____\| \|____\| ጊዜ  አላውቅም --------------------- 99 | | | | |  | |  |
|  | በአሁኑ የእርግዝና ወቅት፣ የቅድመ ወሊድ ክትትል ያገኘሽው ከየት ነበር? | | - - - 1. ሆስፒታል       2. የጤና ጣቢያ ማዕከል       3. የግል ሆስፒታል / ክሊኒክ       4. ሌላ (ዝርዝር ይግለጹ)__________ | | | | | | | |  |
|  | በዚህ የእርግዝና ወቅት፣ የቴታነስ መከላከያ ክትባት በክንድሽ ተሰጥቶሽ ያውቃል? | | 1. አዎ 2. የለም | | | | | |  | |  |
|  | በዚህ የእርግዝና ወቅት የቴታነስ መከላከያ ክትባት በክንድሽ ተሰጥቶሽ የሚያውቅ ከሆነ፣ በዚህ እርግዝና ወቅት ቲታነስ መርፌ ለምን ያህል ጊዜ ነው የወሰድሽው? | | _____________ ጊዜ | | | | | |  | |  |
|  | በዚህ እርግዝና ወቅት የ” አይረን እና ፎሊክ አሲድ” (ለደም ማነስ ችግርን ለመከላከል የሚወሰድ) እንክብል መድሃኒት አግኝተሻል/ወስደሻል? | | 1. አዎ, ለ _________ ወራት ቆይታ  2. አልወሰድኩም | | | | | |  | |  |
|  | ከአንቺ መኖሪያ ቤት ወደ ጤና ተቋም (የጤና ጣቢያ ወይም ሆስፒታል) ለመሄድ በግምት ምን ያህል ርቀት ይሆናል? | | ያለው ርቀት _________ ኪ. ሜ አላውቅም .........................99 | | | | | |  | |  |
|  | ከአንቺ መኖሪያ ቤት ወደ ጤና ተቋም (የጤና ጣቢያ ወይም ሆስፒታል) በእግር ጉዞ ለመሄድ ምን ያህል ጊዜ ይፈጅብሻል? | | ደቂቃዎች ................................. አላውቅም .........................99 | | | | | |  | |  |
|  | ካሁን በፊት የደም ግፊት ህመም አሞሽ ያውቃል? | | 1. አዎ  2. የለም | | | | | |  | |  |
|  | ከቤተሰቦችሽ መካከል የደም ግፊት ህመም አሞት የሚያውቅ የቤተሰብ አባል አለ? | | 1. አዎ  2. የለም | | | | | |  | |  |
|  | ከቤተሰቦችሽ መካከል የስኳር በሽታ/ ህመም አሞት የሚያውቅ የቤተሰብ አባል አለ? | | 1. አዎ  2. የለም | | | | | |  | |  |
|  | ከቤተሰብ ወደ ቤተሰብ የሚተላለፍ በሽታ/ ተዛማጅ የጤና ሁኔታ አለሽ? | | 1. አዎ፣ ይገለፅ ________________  2. የለም | | | | | |  | |  |
|  | በአሁኑ የእርግዝና ወቅት፣ የወባ በሽታ አሞሽ ነበር? | | 1. አዎ  2. የለም | | | | | |  | |  |
|  | በአሁኑ የእርግዝና ወቅት፣ ከሚከተሉት ችግሮች መካከል የትኞቹ አጋጥሞሻል?   1. ከብልት የሚወጣ የደም ፈሳሽ……………………………………………. 2. ከባድ ራስ ምታት………………………………………………………… 3. ከፍተኛ የማቅለሽለሽ ስሜት ወይም ማስታወክ …………… 4. ከፍተኛ የደም ግፊት…………………………………………………….. 5. ትኩሳት………………………………………………………………………….. 6. የሆድ ቁርጠት…………………………………………………………….. 7. ሌላ (ይገለፅ)_______________________.............................. | | **አዎ የለም**   1. 2 2. 2 3. 2 4. 2 5. 2 6. 2 7. 2 | | | | | |  | |  |
|  | በእርግዘዝና ወቀት ያጋጠመሽን የጤና ችግር ለመቅረፍ ምን አድርገሽ ነበር? | | 1. አልታመምኩም ነበር 2. ምንም 3. በጤና ባለሙያ ህክምና አግኝቻለሁ 4. ባህላዊ ሕክምና ተጠቅሜያለሁ 5. ሌላ, ይገለፅ ____________ | | | | | |  | |  |
|  | በቅድመ ወሊድ እንክብካቤ ክትትል ወቅት ዶክተር፣ ነርስ ወይም ሌላ የጤና ባለሞያ ሠራተኛ ከዚህ በታች የተዘረዘሩትን ነገሮች አነጋግሮሻል?   1. በእርግዝና ወቅት አልኮል መጠጣት ወይም ሲጋራ ማጨስ ልጅሽን ሊጎዳ እንደሚችል……………………………….. 2. ትክክለኛው የወሊድ ቀን ከመድረሱ በፊት ሊያጋጥም ስለሚችለው የወሊድ ምጥ/ያለጊዜው የሚመጣ ምጥ ምልክቶች…………………………………………………………………………………………………………………………………. 3. በእርግዝና ወቅት ሊያጋጥሙ ስለሚችሉ ችግሮች እና ምልክቶቻቸው……………………………………………………. 4. የወሊድ ምጥ ቀድሞ ቢመጣ ምን ማድረግ እንዳለብሽ……………………………………………………………………… 5. በእርግዝና ወቅት ለሚያጋጥሙ ችግሮች ምን ማድረግ እንዳለብሽ……………………………………………………….. 6. ስለ ኤች አይ ቪ/ኤድስ ምርመራ ማድረግ አስፈላጊነት………………………………………………………………………… 7. ልጅ ለመውለድ የት መሄድ እንዳለብሽ…………………………………………………………………………………………… 8. ከወሊድ በኋላ ስላለው ጊዜ (ለምሳሌ የጡት ወተት ስለማጥባት፣ ስለ አመጋገብ፣ ስለ ልጅ እንክብካቤ)? …… 9. ከወሊድ በኃላ ሊያጋጥም ስለሚችል የስሜት ለውጥ/ የጭንቀት ችግር?………………………………………………. | | | | | **አዎ የለም**   1. 2 2. 2 3. 2 4. 2 5. 2 6. 2 7. 2 8. 2 9. 2 | | | | |  |
|  | በአሁኑ የእርግዝና ወቅት፣ የቅድመ ወሊድ እንክብካቤ ክትትል ካላደረግሽ፣ ያላደረግሽበት ምክንያቱ ምንድን ነው? | 1. እንዲህ ያለ አገልግሎት ስለመኖሩ አላውቅም 2. የጤና ተቋም ከምኖርበት ቤት ስለሚርቅ 3. ምንም ዓይነት የጤና ችግር ስላልነበረኝ 4. የጤና ባለሙያዎች ታካሚዎችን የሚያክሙበት/የሚይዙበትን መንገድ አልወድም 5. እምነቴ ስለማይፈቅድልኝ 6. ሌላ, ይገለፅ ________________ | | | | | | |  | |  |
|  | በአሁኑ የእርግዝና ወቅት፣ የቅድመ ወሊድ እንክብካቤ ክትትል ስታደርጊ ባለቤትሽ/ ጓደኛሽ ወደ ጤና ተቋም እንዲመጣ ተነግሮሽ ነበር? | | | | 1. አዎ  2. የለም | | | |  | |  |
|  | ባለቤትሽ/ጓደናሽ በእርግዝና ክትትል ወቅት አብሮሽ በህክምና ተቋም ተገኝቶ ነበር? | | | | 1. አዎ 2. የለም | | | |  | |  |
|  | በአሁኑ የእርግዝና ወቅት በማንኛውም ጊዜ የባለቤት / የትዳር ጓደኛሽ በጉልበት ገፍቶሽ, በጥፊ/በእርግጫ መትቶሽ, ድብደባ ወይም በሌላ መንገድ አካላዊ ጉዳት አድርሶብሽ ያውቃል? | | | | 1. አዎ  2. የለም | | | |  | |  |
|  | በአሁኑ የእርግዝና ወቅት በማንኛውም ጊዜ የባለቤት / የትዳር ጓደኛሽ በግብረ ሥጋ ግንኙነት የግድ እንድትፈጽሚ ወይም አንቺ በማትፈልጊበት ጊዜ ወሲባዊ ግንኙነት እንድትፈጽሚ አስገድዶሽ ያውቃል? | | | | 1. አዎ  2. የለም | | | |  | |  |
|  | በአሁኑ የእርግዝና ወቅት በማንኛውም ጊዜ፣ የባለቤት / የትዳር ጓደኛሽ ሰድቦሽ፣ አዋርዶሽ፣ ወይም አንቺን ለመጉዳት አስፈራርቶሽ ያውቃል ? | | | | 1. አዎ  2. የለም | | | |  | |  |
|  | በአሁኑ የእርግዝና ወቅት፣ በህመም ምክንያት ለህክምና ሆስፒታል/ጤና ተቋም ገብተሸ ነበር? | | | | 1. አዎ 2. የለም | | | |  | |  |
|  | ለጥያቄ ቁጥር 339 መልስዎ “አዎ” ከሆነ፣ ሆስፒታል/ጤና ተቋም የገባሽበት ህመም/ ምክንያት ምን ነበር? | | | |  | | | |  | |  |
|  | በአሁኑ የእርግዝና ወቅት፣ ለወሊድ ዝግጅት እንዲሆን በእናቶች የወሊድ ማቆያ ክፍል ቆይተሸ ነበር? | | | | 1. አዎ 2. የለም | | | |  | |  |
|  | ለጥያቄ ቁጥር 440 መልስሽ “አዎ” ከሆነ፣ ለምን ያክል ቀናት እና ሰዓታት በእናቶች ማቆያ ክፍል ነበርሽ? | | | | ________________ ቀናት  _________________ሰዓታት | | | |  | |  |
| **ክፍል 5. ስለ ሱስ የሚያስይዙ ነገሮች መጠቀምን የሚመለከቱ ጥያቄዎች** | | | | | | | | | | | |
| አሁን ስለ ሱስ የሚያስይዙ ነገሮች አጠቃቀም አንዳንድ ጥያቄዎችን እጠይቅሻለሁ | | | | | | | | | | | |
| **ተ.ቁ** | **ጥያቄዎች** | | | **ምላሾች** | | | | **Skip to** | | |  |
|  | የአልኮል መጠጦችን ጠጥተሽ ታውቂያለሽ? (ለምሳሌ፣ እንደ ጠላ፣ ጠጅ፣ አረቂ ወይም ቢራ የመሳሰሉትን) | | | 1. በጭራሽ   2. በየቀኑ እጠጣለሁ  3. በሳምንት አንዴ ወይም ሁለት ጊዜ  4. ሌሎች (ይግለጹ) _______  5. መልስ የለም | | | | 503 | | |  |
|  | ካሁን በፊት አልኮል ጠጥተሽ የምታውቂ ከሆነ፣ በአሁኑ የእርግዝና ወቅት የአልኮል መጠጦችን ጠጥተሽ ታውቂያለሽ? (ለምሳሌ፣ እንደ ጠላ፣ ጠጅ፣ አረቂ ወይም ቢራ የመሳሰሉትን) | | 1. አዎ  2. የለም | | | | |  | | |  |
|  | እንደ ሲጋራዎች ያሉ የትንባሆ ምርቶችን አጭሰሽ/ ተጠቅመሽ ታውቂያለሽ? | | | 1. በጭራሽ   2. በየቀኑ እጠቀማለሁ  3. በሳምንት አንዴ ወይም ሁለት ጊዜ  4. ሌሎች (ይግለጹ) _______  5. መልስ የለም | | | | 505 | | |  |
|  | ካሁን በፊት እንደ ሲጋራዎች ያሉ የትንባሆ ምርቶችን አጭሰሽ/ ተጠቅመሽ የምታውቂ ከሆነ፣ በአሁኑ የእርግዝና ወቅት እንደ ሲጋራዎች ያሉ የትንባሆ ምርቶችን ተጠቅመሽ ታውቂያለሽ? | | 1. አዎ  2. የለም | | | | |  | | |  |
|  | ካሁን በፊት ጫት ቅመሽ ታውቂያለሽ? | | | 1. በጭራሽ   2. በየቀኑ እቅማለሁ  3. በሳምንት አንዴ ወይም ሁለት ጊዜ  4. ሌሎች (ይግለጹ) _______  5. መልስ የለም | | | | 507 | | |  |
|  | ካሁን በፊት ጫት ቅመሽ የምታውቂ ከሆነ፣ በአሁኑ የእርግዝና ወቅት ጫት ቅመሽ ታውቂያለሽ? | | - - - 1. አዎ       2. የለም | | | | |  | | |  |
|  | ካሁን በፊት እንደ ሃሺሽ / ሺሻ የመሳሰሉ አደንዛዥ እፆችን/ መድኃኒቶችን ተጠቅመሽ ታውቂያለሽ? | | | 1. በጭራሽ   2. በየቀኑ እጠማለሁ  3. በሳምንት አንዴ ወይም ሁለት ጊዜ  4. ሌሎች (ይግለጹ) _______  5. መልስ የለም | | | | **601** | | |  |
|  | ካሁን በፊት እንደ ሃሺሽ / ሺሻ የመሳሰሉ አደንዛዥ እፆችን/ መድኃኒቶችን ተጠቅመሽ የምታውቂ ከሆነ፣ በአሁኑ የእርግዝና ወቅት ሃሺሽ / ሺሻ የመሳሰሉ አደንዛዥ መድኃኒቶችንተጠቅመሽ ታውቂያለሽ? | | 1. አዎ 2. የለም   2. | | | | |  | | |  |

**ስለትብብርዎ በጣም አመሰግናለሁ!!**
